# Supplementary material for: Design of hidden thermodynamic driving for non-equilibrium systems via mismatch elimination during DNA strand displacement
Source: Nat Commun. 2020 May 22;11:2562. doi: 10.1038/s41467-020-16353-y (PMC7244503; doi:10.1038/s41467-020-16353-y)
Supplement: Supplementary file 3 — Supplementary Information [file 41467_2020_16353_MOESM3_ESM.pdf]

Supplementary Material for "Design of hidden thermodynamic driving for non-equilibrium systems via mismatch elimination during DNA strand displacement"

Natalie E. C. Haley[1], Thomas E. Ouldridge *[2], Ismael Mullor Ruiz[3], Alessandro Geraldini[3], Ard A. Louis[3], Jonathan Bath[1], and Andrew J. Turberfield †[1]

[1] *Clarendon Laboratory, Department of Physics, University of Oxford, Parks Road, Oxford, OX1 3PU, United Kingdom.*
[2] *Imperial College Centre for Synthetic Biology and Department of Bioengineering, Prince Consort Road, Imperial College London, London SW7 2AZ, United Kingdom.*
[3] *Rudolf Peierls Centre for Theoretical Physics, Department of Physics, University of Oxford, Keble Road, OX1 3NP*

April 2, 2020

Contents

1 Supplementary Note 1: Additional data **3**
1.1 Reporter properties . . . 3
1.1.1 Speed of reporter response . . . 3
1.1.2 Leak reactions for defects at positions 15-17 observed using reporter A . . 4
1.1.3 Comparison of reporter complexes . . . 6
1.2 Simulation of mismatch elimination under first-order conditions . . . 6
1.3 Further data on mismatch elimination in a catalytic motif . . . 7
1.3.1 Second replica of experiments showing real-time response of catalyst-triggered and non-triggered systems . . . 7
1.3.2 Late addition of reporters . . . 8
1.4 Further data on two-toehold mismatch elimination, and comparison to an alternative pulse-generating system . . . 9

2 Supplementary Note 2: Sequences used for experiments **14**
2.1 O, T, I strands . . . 14
2.2 Reporters for mismatch position-dependent measurements . . . 16
2.3 Strands used in the minimal catalytic motif . . . 16
2.4 Two-toehold sequences . . . 16

3 Supplementary Note 3: Data Processing and Fitting **18**
3.1 Basic mismatch elimination following single-toehold invasion . . . 18
3.1.1 Half completion time . . . 18
3.1.2 Second-order fit with exponential decay . . . 18
3.1.3 Second-order fit data up to 99% peak intensity . . . 18

*t.ouldridge@imperial.ac.uk
†andrew.turberfield@physics.ox.ac.uk

3.2 Fitting the kinetics of the two-toehold system 20
3.3 All fitted curves obtained from single experiments used to produce the experimental points for single-toehold displacement 22
3.3.1 Position 2 22
3.3.2 Position 3 23
3.3.3 Position 4 24
3.3.4 Position 5 25
3.3.5 Position 6 26
3.3.6 Position 7 27
3.3.7 Position 8 28
3.3.8 Position 9 29
3.3.9 Position 10 30
3.3.10 Position 11 31
3.3.11 Position 12 32
3.3.12 Position 13 33
3.3.13 Position 15 33
3.3.14 Position 17 35
3.3.15 No Mismatch 36
3.4 Unprocessed data for experiments on the minimal catalytic motif 37
3.4.1 Unprocessed data for experiments monitored with a reporter in real time 37
3.4.2 Unprocessed data for experiments involving late addition of the reporter . 37
3.5 Unprocessed data from single experiments for two-toehold displacement 40
3.5.1 Unprocessed data for two-toehold experiments with mismatches kinetically favouring I_3 40
3.5.2 Unprocessed data for two-toehold experiments with mismatches kinetically favouring I_5 41
3.5.3 Unprocessed data for mismatch-free invasion in the two-toehold system . 42
3.5.4 Unprocessed data for the two-toehold system with reversed displacement domains 43

4 Supplementary Note 4: Simulation protocols and data 45
4.1 Forward flux sampling to obtain displacement rates 45
4.2 Protocols and detailed results for kinetic measurements 45
4.3 Umbrella sampling to obtain free energies of reaction and free-energy profiles . . 49
4.4 Protocols for thermodynamic sampling 50

1 Supplementary Note 1: Additional data

1.1 Reporter properties

1.1.1 Speed of reporter response

The fluorescence output signal triggered by addition of 12.5 nM output strand of three different types to 25 nM Reporter A is shown in Supplementary Figure 1. Half completion time estimates from this data suggest a second-order rate constant of $\sim 2 \times 10^6$ $\mathrm{M}^{-1}s^{-1}$ for the reporter system which is approximately 10 fold greater than the most rapid reaction rate constant fitted. We therefore conclude that our assumption of a rapid reporter reaction is valid.

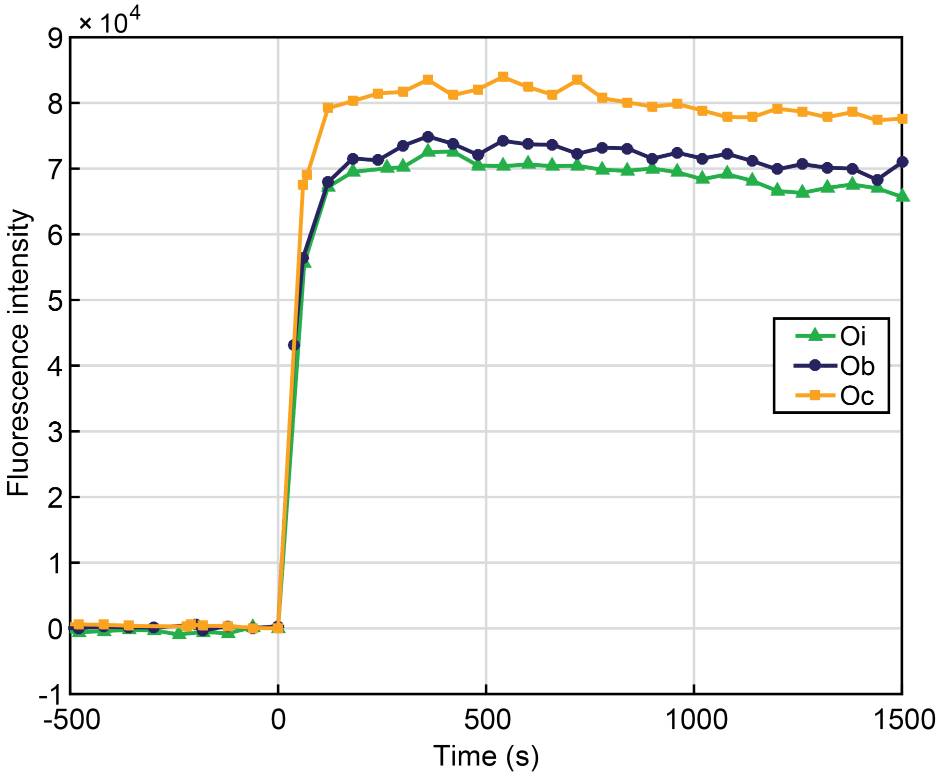

Figure 1: Reporter A triggered by addition of three different free output strands at t=0 (each curve represents a single experiment). In each case the fluorescence output signal increases rapidly and reaches half maximum at approximately 40 s.

1.1.2 Leak reactions for defects at positions 15-17 observed using reporter A

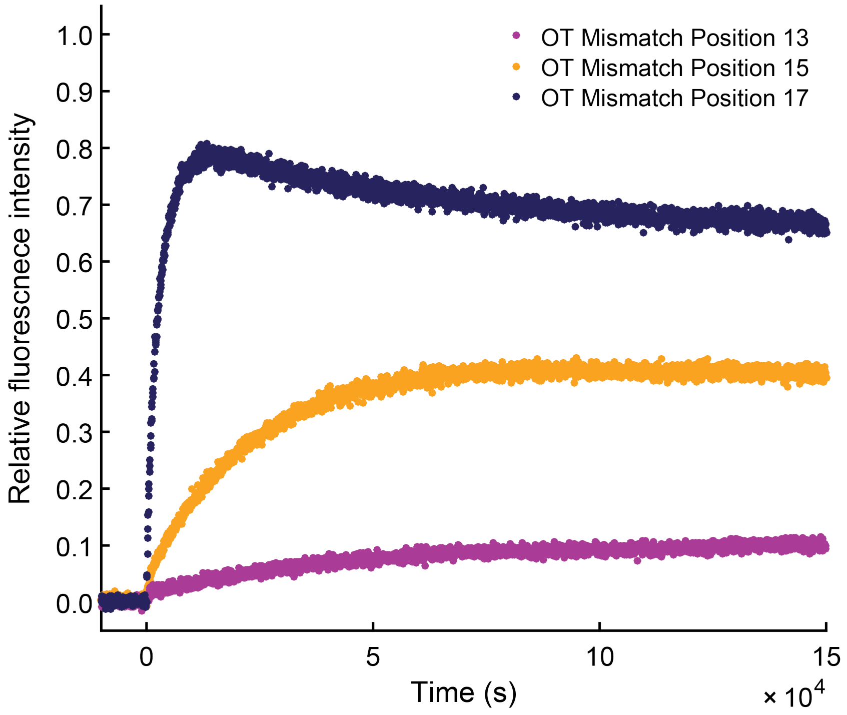

Figure 2: Leak reactions between Reporter A and OT complexes with mismatches at positions 13, 15 and 17. 12.5 nM OT was added at $t = 0$ to 25 nM Reporter A (each curve represents a single experiment). Data was normalised by first subtracting a background corresponding to the fluorescence intensity at the point of OT addition, then dividing by half the fluorescence increase produced by adding a large excess (125 nM) of O. This value is approximately equal to the maximum increase in fluorescence achievable by addition of 12.5 nM (0.5 equivalents) of O. Significant leak reactions occur for defect positions 17 and 15, but not for position 13.

In Supplementary Figure 2, we present evidence that OT duplexes with mismatches at positions 15 and 17, but not earlier mismatches, were able to trigger leak reactions without the presence of I at a significant rate when using reporter A. We hypothesise that this leak reaction is due to occasional spontaneous fraying of the OT duplex, revealing part of the toehold on O that can bind to the reporter. We design an alternative reporter system for which the complementary toehold on O is buried deeper within the initial duplex, eliminating this problem. In Supplementary Figure 3 we demonstrate that this second reporter is also rapid enough relative to the reactions of interest to be treated as instantaneous. In Supplementary Figure 4 we show that the second reporter successfully suppresses reporter-induced leak reactions at positions 15 and 17.

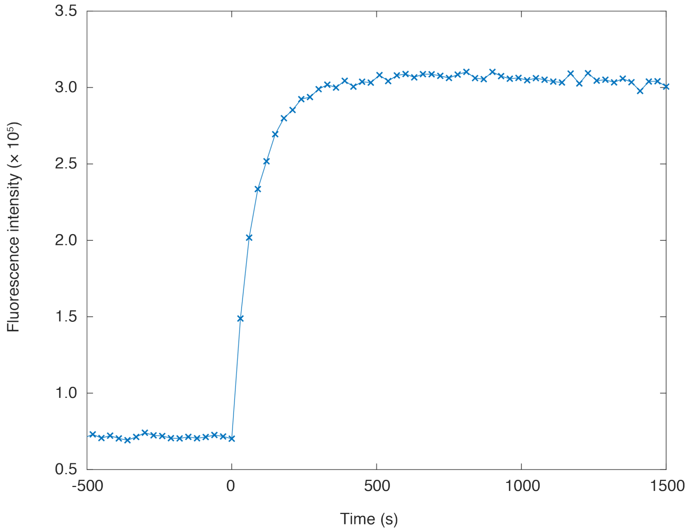

Figure 3: Reporter B triggered by addition of output strand Oa at t=0 (curve represents a single experiment). The fluorescence output signal increases rapidly and reaches half maximum at approximately 60 s.

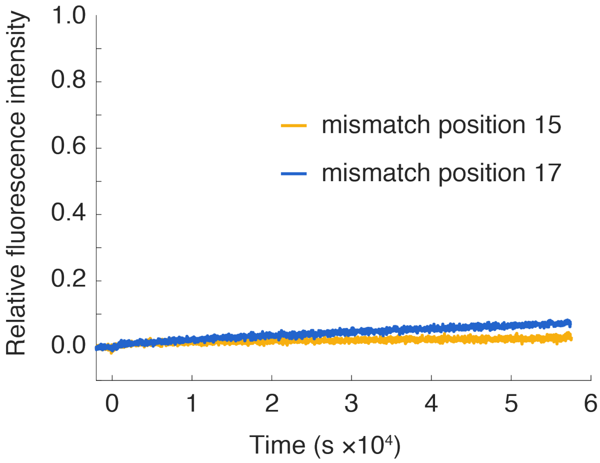

Figure 4: Leak reactions between Reporter B and OT complexes with mismatches at positions 15 and 17. 12.5 nM OT was added at $t = 0$ to 25 nM Reporter B (each curve represents a single experiment). Data was normalised by first subtracting a background corresponding to the fluorescence intensity at the point of OT addition, then dividing by half the fluorescence increase produced by adding a large excess (125 nM) of O. This value is approximately equal to the maximum increase in fluorescence achievable by addition of 12.5 nM (0.5 equivalents) of O. No significant leak reactions occur for either position 17 or position 15.

1.1.3 Comparison of reporter complexes

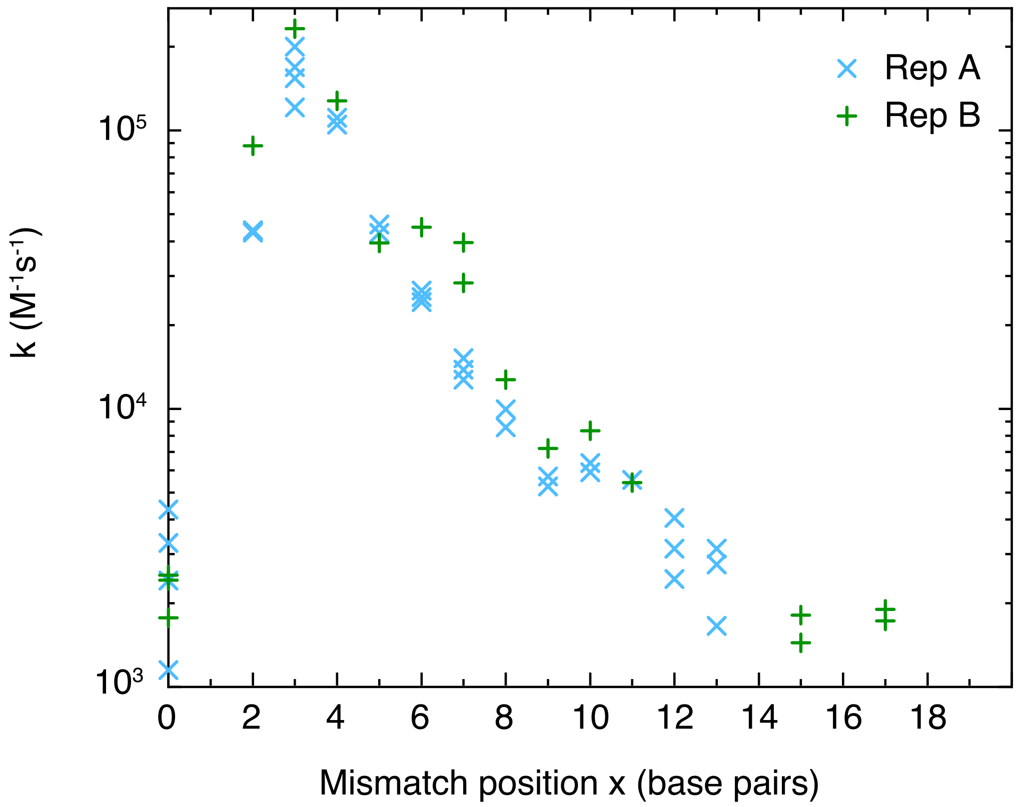

Figure 5: Fitted rate constants from individual experiments using two different reporter complexes: A (dark blue $\times$), and B (pale blue $+$). Both reporter complexes can be used to measure the perfectly matching case and mismatches up to position 11, however only Reporter A can be used to measure positions 12 and 13 due to mismatches with the Reporter B toehold sequence, and only Reporter B can be used to measure positions 15 and 17 due to leak reactions with reporter A (see Supplementary Note 1.1.2). Data was fitted from the start of the reaction to the time at which 99% of the peak intensity was achieved. Both reporters were used to generate data fitted to produce Fig. 2 (d) of the main text.

To alleviate the problem outlined in Supplementary Note 1.1.2, we introduce a second reporter B that couples to a toehold more deeply buried in the initial OT duplex. In Fig. 5, we present evidence that fits to data recorded using both reporter complexes A and B produce similar fitted rate constants for those mismatch positions that can be studied with both, supporting their use in a combined set of results.

1.2 Simulation of mismatch elimination under first-order conditions

Encouraged by the success of oxDNA in reproducing these experimental data, here we use the model to probe mismatch-elimination TMSD under conditions in which the strand-displacement reaction is first-order, conditions that were not studied in the experiments reported. In the limit of high $[I]$ the toehold is essentially always occupied and the reaction is first order with a rate given by $k \approx 1/\tau$, where τ is the average time spent in the three-stranded complex per successful reaction. This time can be directly inferred from trajectories launched from the toehold-bound

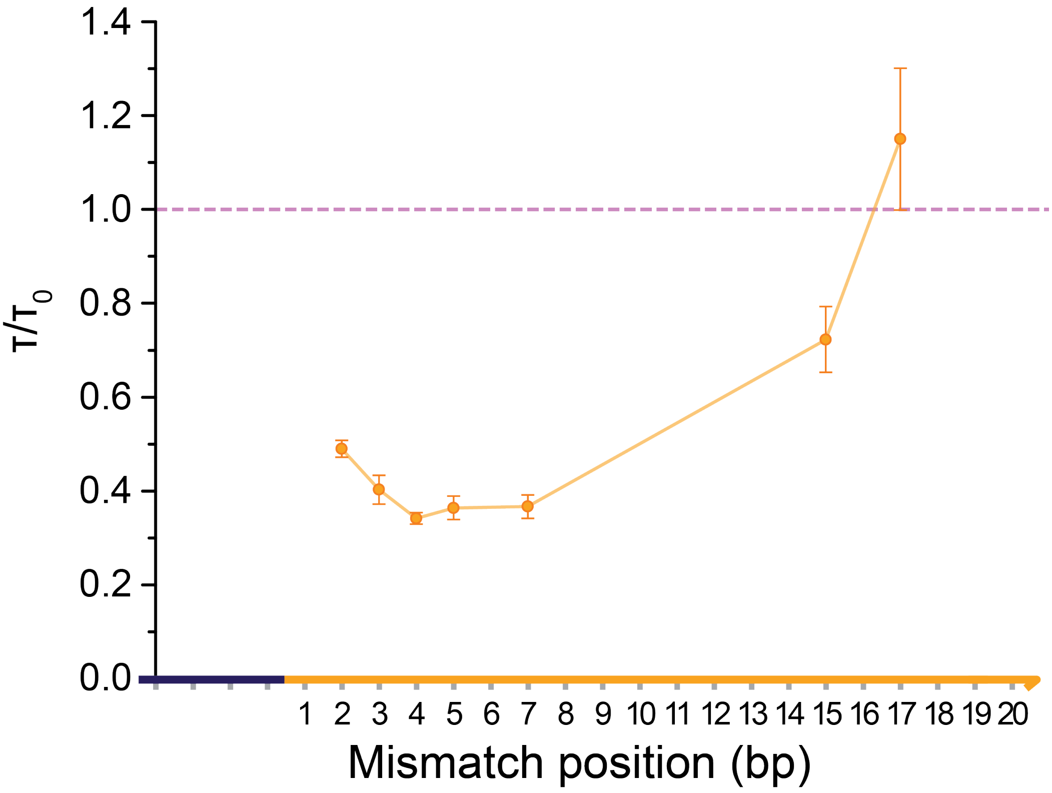

Figure 6: Average time τ spent in the three-stranded complex per successful displacement event, as a function of mismatch position x, relative to the mismatch-free case. Error bars show SEM for $n = 6$, and do not include the (fixed) contribution to the error on the ratio from the error for the mismatch-free case, which is $\sim \pm 0.12$ ($n = 6$).

state (Supplementary Note 4.2) for which simulation results are plotted in Supplementary Figure 6. oxDNA predicts that it is possible to enhance displacement in the first order limit by a factor of ~ 3 through early mismatch elimination.

Interestingly, the optimal speed-up is for mismatch sites slightly further into the duplex than in the second-order limit, because guaranteeing a high probability of successful branch migration is not the same thing as making branch migration as fast as possible when it does happen. If branch migration were modelled as a simple, unbiased random walk,[1] then the optimal speed-up in the first-order limit would arise from a defect in the centre of the displacement domain, splitting the random walk in half and halving the expected reaction time. We see a faster speed-up, and an optimal defect position nearer the toehold than the centre of the duplex, consistent with the presence of an initial barrier to displacement[1] that can be alleviated by early mismatches.

1.3 Further data on mismatch elimination in a catalytic motif

1.3.1 Second replica of experiments showing real-time response of catalyst-triggered and non-triggered systems

The experiment reported in Fig. 5 of the main text was repeated using strands that were re-diluted from the IDT-supplied stock. The results, plotted in Supplementary Figure 7, show the same features. Systems with a designed thermodynamic drive – either from mismatch elimination or the use of a longer fuel strand C_2 – exhibit more effective production of reporter-triggering output $B/B_{\mathrm{TT}}/B_{\mathrm{CA}}$. However, the longer fuel strand C_2 also gives rise to a substantial leak reaction that is much less of a problem for the mismatch-elimination systems. Unprocessed data

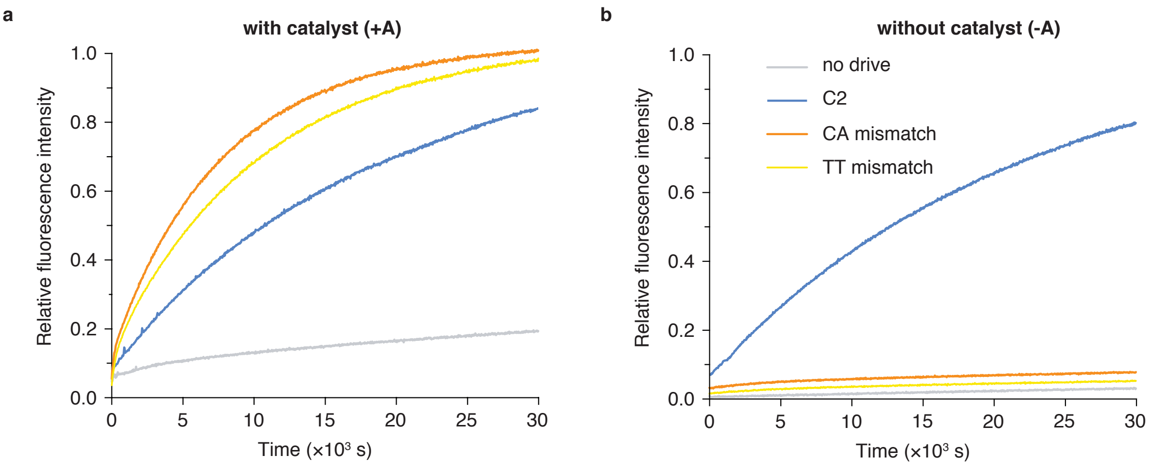

Figure 7: Replica of the experiments reported in Fig. 5 of the main text (each curve represents a single experiment). (a) Relative fluoresence intensity of a reporter for single-stranded B after 40 nM of BD is mixed with 200 nM of C and triggered by 5nM of the catalyst strand A. Plotted are curves for the default system in which BD and CD have the same number of base pairs, and three variants in which the catalytic exchange has a thermodynamic drive: one with a variant strand C_2 that can form two additional base pairs with D, and systems in which variant A and B strands form C-A and T-T mismatches with D that are eliminated in the CD duplex. Systems with thermodynamic drive show strongly enhanced reaction rate and yield relative to the default. (b) Monitoring of leak reactions in systems that are equivalent to (a), but without the addition of the catalyst strands. The mismatch-elimination systems show much lower rates of leak reaction than the C_2 variant.

corresponding to this experiment are reproduced in Supplementary Figure 31, with unprocessed data corresponding to Fig. 5 of the main text in Fig. 30.

1.3.2 Late addition of reporters

Additional experiments were performed to probe the behaviour of the catalytic motif without the reporter complex acting as a sink for the output strand. Here, reporter complexes were added three and six hours after mixing all other strands. At this point, free output $B/B_{\mathrm{TT}}/B_{\mathrm{CA}}$ molecules quickly generate fluorescent output by displacing the locking strand from the reporter complex. The size of the sudden jump in fluorescence in Supplementary Figure 8 is therefore indicative of the degree of completion of the reaction prior to the introduction of the reporters. The low completion level of the system without thermodynamic drive is in stark contrast to the essentially 100% completion of the system driven by C-A mismatch elimination. The T-T mismatch system and the system driven with an extended C, strand C_2 show intermediate behaviour.

Oligonucleotides were prepared exactly as for the experiments in Fig. 5 of the main text, except that a working concentration of 100 nM of the catalyst strands $A/A_{\mathrm{TT}}/A_{\mathrm{CA}}$ was prepared by adding 10 µL of the intermediate concentration (1 µM) to 90 µL of hybridization buffer.

Wells were filled with 151 µL of hybridization buffer, 8 µL of the working concentration of $BD/B_{\mathrm{TT}}D/B_{\mathrm{CA}}D$, 1 µL of the corresponding fuel C or C_2 and then 20 µL of the corresponding catalyst $A/A_{\mathrm{TT}}/A_{\mathrm{CA}}$. Well contents were mixed with a pipette and left to react for the corresponding time. The three and six-hour experiments were done simultaneously in the same plate, with the three-hour experiments initiated at the midpoint of the six-hour experiments. After six hours, 20 µL of the working concentration of the reporter complex was added simultaneously in all wells using the multichannel pipette. Fluorescence was recorded for 500 minutes, after which

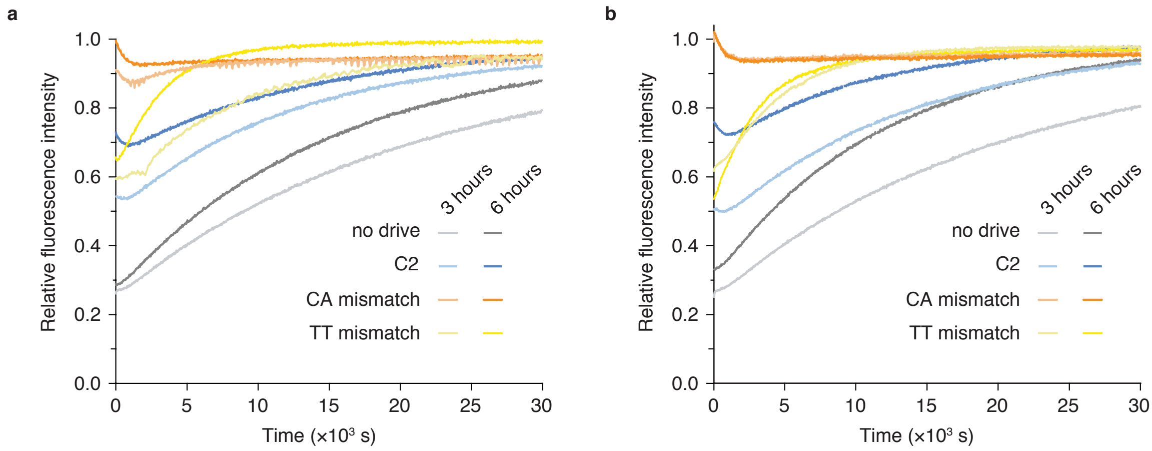

Figure 8: (a) and (b) show the relative fluorescent intensity of a reporter in two replicas of experiments in which the catalysis reaction was left to run for three and six hours before addition of the reporter complex. Each curve represents a single experiment. The initial point reached immediately after reporter addition indicates the level of turnover prior to addition of the reporter; the slow growth towards 100% completion is the continued turnover due to the reporter complex acting as a sink for $B/B_{\rm TT}/B_{\rm CA}$ strands. Experiments were initiated with 11.1 nM of $A/A_{\rm TT}/A_{\rm CA}$, 44.4 nM of $BD/B_{\rm TT}D/B_{\rm CA}D$ and 55.7 nM of C/C_2.

an excess of input (4 µL of the standard catalyst A at 100 µM) was added in order to check the endpoint of the experiment and allow for the normalisation of data. Controls containing only the reporter complex were also included to set a baseline for the experiment.

Data were processed exactly as for Fig. 5 of the main text, except there is no $F_{\rm min}$ baseline for each experiment because the reporter is added after the reaction. We therefore use the average signal of the controls as a baseline to estimate $F_{\rm min}$, ignoring the first 2000 s of the experiment. Processed data for these two replicas are reported in Supplementary Figure 8 and unprocessed fluorescence data in Supplementary Figure32.

1.4 Further data on two-toehold mismatch elimination, and comparison to an alternative pulse-generating system

In Fig. 6 of the main text we present data on a two-toehold system in which a mismatch is located close to the 3' toehold. Invader I_3 is kinetically favoured by this early mismatch elimination, despite a higher concentration of I_5. The rate constants obtained by fitting a second-order kinetics model, outlined in Supplementary Note 3.2 to these data are given in Supplementary Table 1. Also shown in Supplementary Table 1 and Supplementary Table 2 are the results of similar fits to complementary experiments involving: mismatch elimination at the other end of the initial OT duplex (close to the 5' toehold) and an opposite concentration imbalance; mismatch *introduction* at either end of the duplex; and a mismatch-free system initiated with both possible concentration imbalances. Sequences for these experiments are given in Supplementary Note 2.4, raw data in Supplementary Note 3.5, and processed fits are shown in Supplementary Figure 10 and Supplementary Figure 11 as well as Fig. 6 of the main text.

We first note that, in all experiments, the rate constants k_{53} and k_{35} for exchange of I_3 and I_5 i.e. invasion by I_3 of the I_5T duplex and vice versa are very similar to each other. Thus the overall ΔGs for invasion of the OT duplex by I_3 and I_5 are very similar. However, by comparing rates for mismatch elimination with rates for mismatch-free systems, we see that in all cases rates are strongly enhanced by early mismatch elimination (by factors of approximately

10 and 20), whereas late mismatch provides only a factor of 2. These data support our earlier conclusions, and indicate that we have successfully implemented hidden thermodynamic driving in the case of late mismatch elimination.

We note that there is an apparent natural kinetic advantage for the 3' invader, even without the inclusion of the mismatch, despite our attempts to make the toeholds as equivalent as possible. This difference on its own can give rise to weak pulse-like behaviour (Fig. 11). We are uncertain as to the physical cause of this difference, which may be due to differences in the fluorophores at the end of the strands, but the result is that our mismatch-based contribution to the kinetic bias either combines with or works against this intrinsic contribution. As a consequence, we see a much larger ratio between rates k_3 and k_5 when the mismatch is near to the 3' end than the 5' end: we observe an extremely sharp pulse in Fig. 6 of the main text, but a much weaker pulse in Supplementary Figure 10. Nonetheless, both experiments are consistent with the understanding outlined in the main text, and we are able to modulate rates and reaction thermodynamics as expected in both cases.

To explore the robustness of the motif, we also collected data on an a second system exhibiting two-toehold mismatch elimination. This second system differs from the first in having a reversed displacement domain: precise sequences are given in Supplementary Note 2.4. We again explored the effects of eliminating a mismatch at 3 and 18 base pairs from the invading toehold. Raw data for these reactions is shown in Supplementary Note 3.5, and processed fits in Supplementary Figure 12 and Supplementary Figure 13. Rate constants obtained by fitting to the data as described in Supplementary Note 3.2 are shown in in Supplementary Table3. Once again, relative to the mismatch-free case, it is clear that mismatch elimination early during the displacement process has a large effect on reaction rates (by a factor of 20 or more), whereas late mismatch elimination only accelerates reactions by approximately a factor of 2.

We have previously shown that mismatch creation during strand displacement is also able to generate non-monotonic behaviour in which the kinetically favoured invader is eventually supressed in equilibrium.[2] Mismatch creation cannot contribute a hidden thermodynamic driving force since the mismatch destablizes the product state, rather than stabilizing it. It is also interesting to note that mismatch introduction leads to extremely broad peaks (Supplementary Figure 9 and Supplementary Figure 10b) with similar rise and fall times, whereas mismatch elimination leads to a much sharper rise and relatively slow decay to equilibrium. Fundamentally, this difference arises because mismatch creation encourages the subsequent invasion by the competitor strand, whereas mismatch elimination does not (mismatch creation also shows more pronounced peaks when the bias generated by mismatches coincides with the natural 3'/5' kinetic asymmetry discussed above.)

	mismatch type	position relative to 5'	3'	k_5 ($M^{-1}s^{-1}$)	k_3 ($M^{-1}s^{-1}$)	k_{53} ($M^{-1}s^{-1}$)	k_{35} ($M^{-1}s^{-1}$)
T–C → T–A	Elimination	18	3	5.7×10^3	1.3×10^5	9.8×10^2	8.9×10^2
C–G → G–G	Introduction	3	18	$9.8{\times}10^{-4\dagger}$	6.9×10^3	9.8×10^2	8.6×10^2
N/A	None	N/A	N/A	2.8×10^3	7.6×10^3	7.3×10^2	8.5×10^2

Table 1: Fitted second-order rate constants for two-toehold reactions with a 4:2:1 ratio of $I_5 : I_3 : OT$ initially present. Fitted curves corresponding to these values are shown in Fig. 6 of the main text, Supplementary Figure 9, and Supplementary Figure 11a. † indicates that the fit is not able to precisely determine this rate constant, only that it is much smaller than the other rate constants involved.

	mismatch type	position relative to 5'	3'	k_5 ($M^{-1}s^{-1}$)	k_3 ($M^{-1}s^{-1}$)	k_{53} ($M^{-1}s^{-1}$)	k_{35} ($M^{-1}s^{-1}$)
C–C → C–G	Elimination	3	18	2.4×10^4	1.4×10^4	7.8×10^2	9.5×10^2
G–C → G–A	Introduction	18	3	2.1×10^3	3.4×10^2	7.3×10^2	9.9×10^2
N/A	None	N/A	N/A	2.6×10^3	7.9×10^3	9.4×10^2	6.6×10^2

Table 2: Fitted second-order rate constants for two-toehold reactions with a 2:4:1 ratio of $I_5 : I_3 : OT$ initially present. Fitted curves corresponding to these values are shown in Supplementary Figure 10 and Supplementary Figure11b.

	mismatch type	position relative to 5'	3'	k_5 ($M^{-1}s^{-1}$)	k_3 ($M^{-1}s^{-1}$)	k_{53} ($M^{-1}s^{-1}$)	k_{35} ($M^{-1}s^{-1}$)
T–C → T–A	Elimination	3	18	3.6×10^4	5.9×10^3	7.7×10^2	2.4×10^3
C–C → C–G	Elimination	18	3	7.6×10^3	1.1×10^5	7.8×10^2	1.2×10^3
I_5 excess	None	N/A	N/A	2.6×10^3	2.9×10^3	6.3×10^2	1.1×10^3
I_3 excess	None	N/A	N/A	3.0×10^3	2.5×10^3	5.2×10^2	1.6×10^3

Table 3: Fitted second-order rate constants for two-toehold reactions with a reversed displacement domain. Fitted curves corresponding to these values are shown in Supplementary Figure 12 and Supplementary Figure 13.

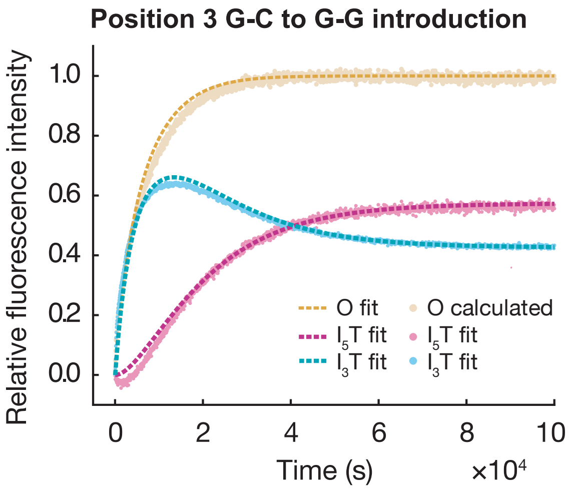

Figure 9: Kinetically favouring I_3 with mismatch creation. Normalized and fitted data (corresponding to fluorescence data of a single experiment, Supplementary Figure 33 (b)) for a kinetic bias introduced by G–C → G–G mismatch introduction 3 bp from the 5' toehold. All reactions have a 4:2:1 ratio of $I_5 : I_3 : OT$ initially present, providing an eventual bias towards the I_5T state. Note that the O curve is inferred from the sum of I_3T and I_5T, with its maximum value used to scale the figure.

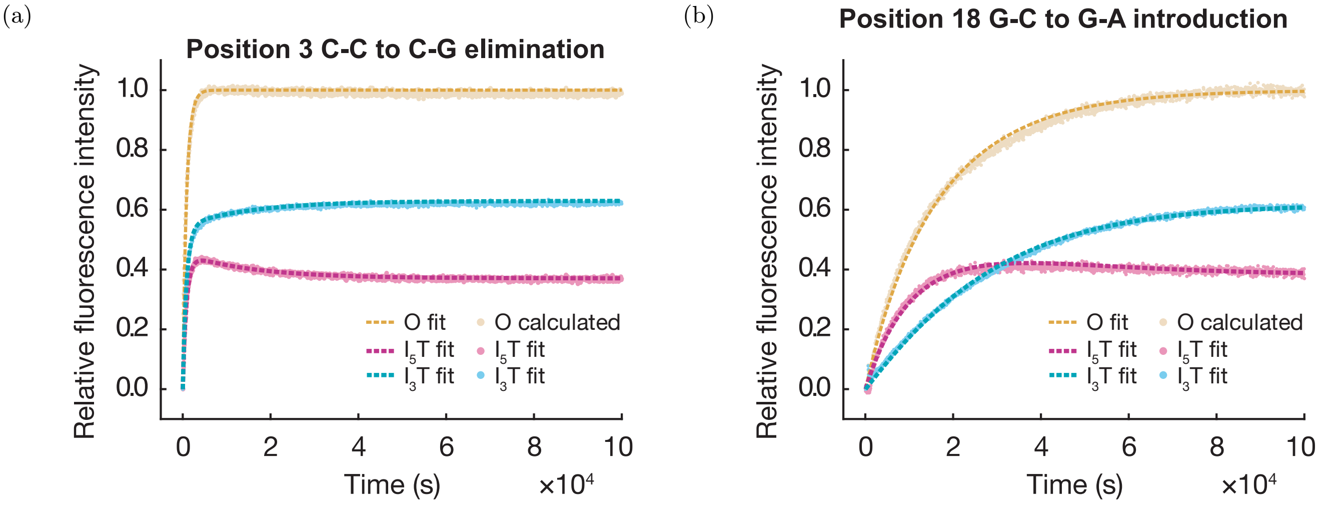

Figure 10: Kinetically favouring I_5 with mismatch position. Normalized and fitted data (corresponding to fluorescence data of a single experiment each, Supplementary Figure 34) for a kinetic bias introduced by C–C → C–G mismatch elimination 3 bp from the 5' toehold (a); or G–C → G–A mismatch introduction 18 bp from the 5' toehold (b). All reactions have a 2:4:1 ratio of $I_5 : I_3 : OT$ initially present, providing an eventual bias towards the I_5T state. Note that the O curve is inferred from the sum of I_3T and I_5T, with its maximum value used to scale the figure.

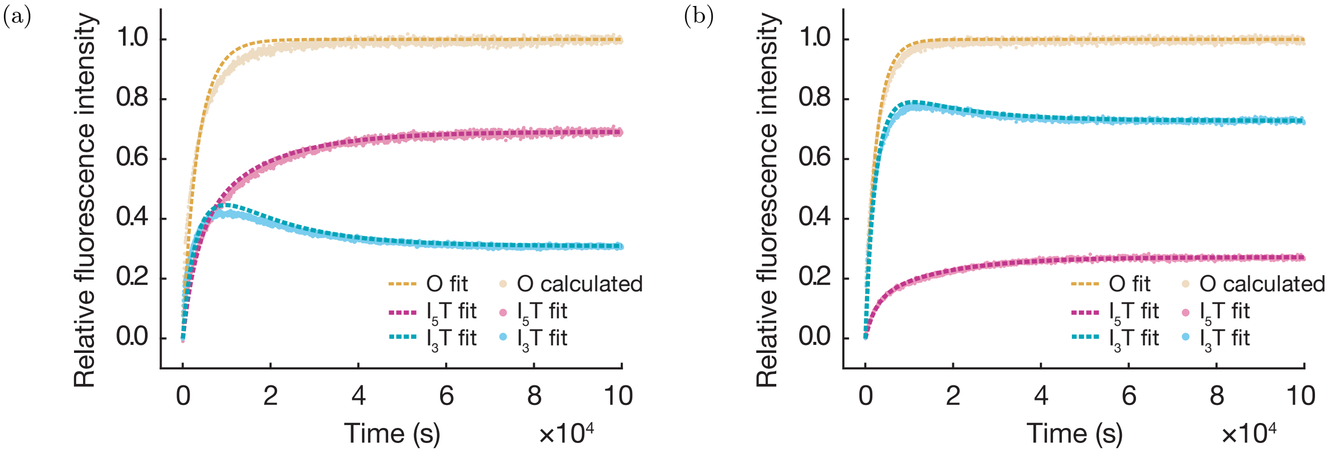

Figure 11: Two-toehold displacement in the absence of mismatches, showing an intrinsic kinetic bias in favour of I_3 in the absence of mismatches. Normalized and fitted data (corresponding to fluorescence data of a single experiment each, Supplementary Figure 35) for two-toehold competitive displacement with no mismatches present in reactant or product duplexes. Reactions with a 4:2:1 ratio of $I_5 : I_3 : OT$ (a) or a 2:4:1 ratio of $I_5 : I_3 : OT$ (b). Note that the O curve is inferred from the sum of I_3T and I_5T, with its maximum value used to scale the figure.

(a) (b)

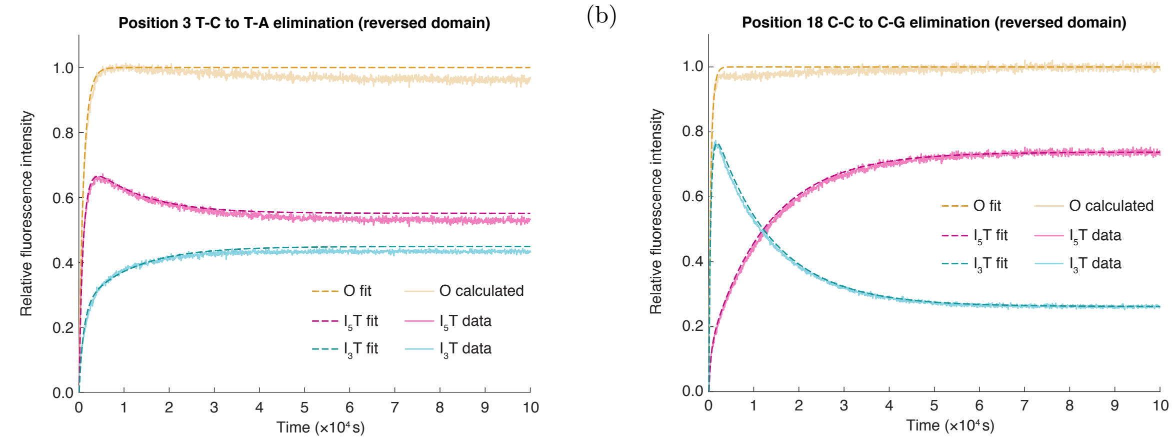

Figure 12: Two-toehold displacement incorporating mismatch elimination with reversed displacement domains. Normalized and fitted data (corresponding to fluorescence data of a single experiment each, Supplementary Figures 36 and 37) for a kinetic bias introduced by T–C $\rightarrow$ T–A mismatch elimination 3 bp from the 5' toehold (a); or C–C $\rightarrow$ C–G mismatch elimination 18 bp from the 5' toehold (b). Both reactions have a concentration bias towards the kinetically disfavoured invader initially present: (a) 2:4:1 ratio of $I_5 : I_3 : OT$, (b) 4:2:1 ratio of $I_5 : I_3 : OT$ Note that the O curve is inferred from the sum of I_3T and I_5T, with its maximum value used to scale the figure.

(a) (b)

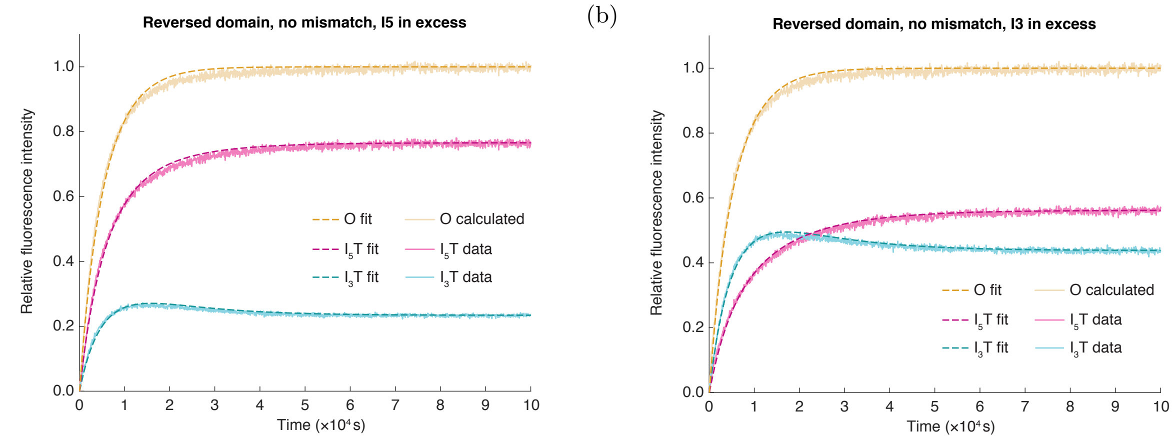

Figure 13: Two-toehold displacement in the absence of mismatches with reversed displacement domains. Normalized and fitted data (corresponding to fluorescence data of a single experiment each, Supplementary Figures 38 and 39) for two-toehold competitive displacement with no mismatches present in reactant or product duplexes. Reactions with a 4:2:1 ratio of $I_5 : I_3 : OT$ (a) or a 2:4:1 ratio of $I_5 : I_3 : OT$ (b). Note that the O curve is inferred from the sum of I_3T and I_5T, with its maximum value used to scale the figure.

2 Supplementary Note 2: Sequences used for experiments

2.1 O, T, I strands

Strand Name	5'–3' Sequence
Oa	ACCACAAACACCCACA CCACTCA AACTCCAACTCAC
Ta_0	TGAT GTGAGTTGGAGTT TGAGTGG
Ia_0	CCACTCA AACTCCAACTCAC ATCA
Ta_5C	TGAT GTGACTTGGAGTT TGAGTGG
Ia_5G	CCACTCA AACTCCAAGTCAC ATCA
Ta_15C	TGAT GTGAGTTGGAGTT TCAGTGG
Ia_15G	CCACTGA AACTCCAACTCAC ATCA
Ta_17C	TGAT GTGAGTTGGAGTT TGACTGG
Ia_17G	CCAGTCA AACTCCAACTCAC ATCA
Ob	ACCACAAACACCCACA CCACTCA AACTCCACACACT
Tb_2C	TGAT ACTGTGTGGAGTT TGAGTGG
Ib_2G	CCACTCA AACTCCACACAGT ATCA
Oc	ACCACAAACACCCACA CCACTCA AACTCCACAACTC
Tc_3C	TGAT GACTTGTGGAGTT TGAGTGG
Ic_3G	CCACTCA AACTCCACAAGTC ATCA
Od	ACCACAAACACCCACA CCACTCA AACTCCACACTAC
Td_4C	TGAT GTACTGTGGAGTT TGAGTGG
Id_4G	CCACTCA AACTCCACAGTAC ATCA
Oe	ACCACAAACACCCACA CCACTCA AACTCCACTACAC
Te_6C	TGAT GTGTACTGGAGTT TGAGTGG
Ie_6G	CCACTCA AACTCCAGTACAC ATCA
Of	ACCACAAACACCCACA CCACTCA AACTCACTCACAC
Tf_7C	TGAT GTGTGACTGAGTT TGAGTGG
If_7G	CCACTCA AACTCAGTCACAC ATCA
Og	ACCACAAACACCCACA CCACTCA AACTACTCCACAC
Tg_8C	TGAT GTGTGGACTAGTT TGAGTGG
Ig_8G	CCACTCA AACTAGTCCACAC ATCA
Oh	ACCACAAACACCCACA CCACTCA AACACTTCCACAC
Th_9C	TGAT GTGTGGAACTGTT TGAGTGG
Ih_9G	CCACTCA AACAGTTCCACAC ATCA
Oi	ACCACAAACACCCACA CCACTCA AAACTCTCCACAC
Ti_10C	TGAT GTGTGGAGACTTT TGAGTGG
Ii_10G	CCACTCA AAAGTCTCCACAC ATCA
Oj	ACCACAAACACCCACA CCACTCA AACTACTCCACAC
Tj_11C	TGAT GTGTGGAGTACTT TGAGTGG
Ij_11G	CCACTCA AAGTACTCCACAC ATCA
Ok	ACCACAAACACCCACA CCACTCA ACTAACTCCACAC
Tk_12C	TGAT GTGTGGAGTTACT TGAGTGG
Ik_12G	CCACTCA AGTAACTCCACAC ATCA
Ol	ACCACAAACACCCACA CCACTCA CTAAACTCCACAC
Tl_13C	TGAT GTGTGGAGTTTAC TGAGTGG
Il_13G	CCACTCA GTAAACTCCACAC ATCA

Table 4: Sequences used for the single-toehold experiments on mismatch elimination. Mismatch regions are underlined.

2.2 Reporters for mismatch position-dependent measurements

Strand Name	5'–3' Sequence	5' Modification	3' Modification
Reporter A output	AC ACCACAAACACCCACA	Cy5	
Reporter A target	TGAGTGG TGTGGGTGTTTGTGGT GT		IowaBlackRQ
Reporter B output	ACCACAAACACCCACA CC	Cy5	
Reporter B target	TT TGAGT GG TGTGGGTGTTTGTGGT		IowaBlackRQ

Table 5: Reporter complex DNA sequences.

2.3 Strands used in the minimal catalytic motif

Strand Name	5'–3' Sequence
Substrate D	GAGTGG GCGAAGGGTTCTTGGAGGAG AG GGTC GG
Output B	GTCCCTATTACCCTTG CC GACC CT CTCCTCCAAGAACCCTTCGC
Output B_{CA}	GTCCCTATTACCCTTG CC GACC CT CTCCTCCAAAAACCCTTCGC
Output B_{TT}	GTCCCTATTACCCTTG CC GACC CT CTCCTCCATGAACCCTTCGC
Catalyst A	CT CTCCTCCAAGAACCCTTCGC CCACTC
Catalyst A_{CA}	CT CTCCTCCAAAAACCCTTCGC CCACTC
Catalyst A_{TT}	CT CTCCTCCATGAACCCTTCGC CCACTC
Fuel C	CC GACC CT CTCCTCCAAGAACCCTTCGC
Fuel $C2$	CC GACC CT CTCCTCCAAGAACCCTTCGC CC
Reporter target	AG GGTC GG CAAGGGTAATAGGAC TTT - Cy3
Reporter lock	Iowa Black FQ - TTT GTCCCTATTACCCTTG CC

Table 6: Strands use in the minimal catalytic motif.

2.4 Two-toehold sequences

Strand Name	5'–3' Sequence	5' Modification	3' Modification
I_5_3G_18A	TC CCACTCAAACTCCACAAGTC GAAG CT	Cy5	IowaBlackRQ
I_3_3G_18A	TC GAAG CCACTCAAACTCCACAAGTC CT	IowaBlackFQ	Cy3
For position 18 T–C ⟶ T–A elimination (kinetically favouring I_3)			
T2c_3C_18T	CTTC GACTTGTGGAGTTTGAGTGG CTTC		
O2c_3G_18C	CCCCTCAAACTCCACAAGTC		
For position 3 C–G ⟶ G–G introduction (kinetically favouring I_3)			
T2c_3G_18T	CTTC GAGTTGTGGAGTTTGAGTGG CTTC		
O2c_3C_18A	CCACTCAAACTCCACAACTC		
For position 3 C–C ⟶ C–G elimination (kinetically favouring I_5)			
T2c_3C_18T	CTTC GACTTGTGGAGTTTGAGTGG CTTC		
O2c_3C_18A	CCACTCAAACTCCACAACTC		
For position 18 G–C ⟶ G–A introduction (kinetically favouring I_5)			
T2c_3C_18G	CTTC GACTTGTGGAGTTTGAGGGG CTTC		
O2c_3G_18C	CCCCTCAAACTCCACAAGTC		
Mismatch free (no kinetic bias intended)			
T2c_3C_18T	CTTC GACTTGTGGAGTTTGAGTGG CTTC		
O2c_3G_18A	CCACTCAAACTCCACAAGTC		
**Reversed displacement domain**			
I_5_3A_18Gr	TC CTGAACACCTCAAACTCACC GAAG CT	Cy5	IowaBlackRQ
I_3_3A_18Gr	TC GAAG CTGAACACCTCAAACTCACC CT	IowaBlackFQ	Cy3
For position 3 T–C ⟶ T–A elimination (kinetically favouring I_5)			
T2r_3T_18C	CTTC GGTGAGTTTGAGGTGTTCAG CTTC		
O2r_3C_18G	CTGAACACCTCAAACTCCCC		
For position 18 C–C ⟶ C–G elimination (kinetically favouring I_3)			
T2r_3T_18C	CTTC GGTGAGTTTGAGGTGTTCAG CTTC		
O2r_3A_18C	CTCAACACCTCAAACTCACC		
Mismatch free (no kinetic bias intended)			
T2r_3T_18C	CTTC GGTGAGTTTGAGGTGTTCAG CTTC		
O2r_3A_18G	CTGAACACCTCAAACTCACC		

Table 7: Sequences for two-toehold experiments. Mismatch positions are quoted relative to the 5' end of the T strand.

3 Supplementary Note 3: Data Processing and Fitting

3.1 Basic mismatch elimination following single-toehold invasion

Data were zeroed by setting t=60 at the first data point collected after mixing (60s is the measured typical mixing time) and subtracting from all fluorescence counts the fluorescence of the last data point collected before addition of invader. Raw fluorescence traces exhibit varying degrees of drift and/or photobleaching over the course of the experiment. To ensure that the reported data are not unduly influenced by this effect, three methods for extracting the second-order rate constant from the resultant data were tested. The consistency of these methods, which compensate for the drift/photbleaching in different ways, is demonstrated in Supplementary Figure 14. For the second-order methods, processed curves were fitted using MATLAB function '`fit`'.

3.1.1 Half completion time

The relationship between second-order rate constant k and time until half completion $t_{1/2}$ for a second-order reaction with stoichiometric initial concentrations c_0 is given by Supplementary Equation 1:

$$k = \frac{1}{c_0 \times t_{1/2}} \tag{1}$$

Using this relationship, values of k were determined by finding the time at which data, smoothed by a rolling average window of 5 data points, exceeds half the maximum value. If the first data point exceeding half the maximum fluorescence differs from half the maximum fluorescence by more than 1% then the half completion time is found by linear interpolation. Results are shown in dark blue circles in Supplementary Figure 14a, and example fits are given as dark blue lines in Supplementary Figures 14b and 14c.

3.1.2 Second-order fit with exponential decay

Photobleaching was observed in many of the samples which varied from sample to sample as a result of factors such as fluorimeter bulb age/ illumination slit size, and amount of air trapped in the cuvette. To account for the effect of these bleaching effects the second-order fitting function was modified with an exponential decay envelope:

$$ae^{-bt}\left(1 - \frac{1}{1 + c_0 kt}\right) \tag{2}$$

The three parameters, a, k, and b were fitted and results for fitted k are shown as green diamonds in Supplementary Figure 14a. Example fits are given as solid green lines in Supplementary Figure 14b and 14c. The dotted green lines in Supplementary Figures 14b and 14c show the second-order function without exponential decay envelope ($b = 0$) for parameters a and k obtained from fits using the envelope as described above for comparison to the other fit methods.

3.1.3 Second-order fit data up to 99% peak intensity

This is the method used to produce the fits used in the main text. Rather than attempt to compensate for the experimentally observed decay of fluorescence signal at long times, we fit all of the data points up to the time at which the first data point exceeds 99% the maximum recorded fluorescence value and ignore later data points for purposes of the fit. The fitted function is given by Supplementary Equation 3:

$$a\left(1 - \frac{1}{1 + c_0 kt}\right) \tag{3}$$

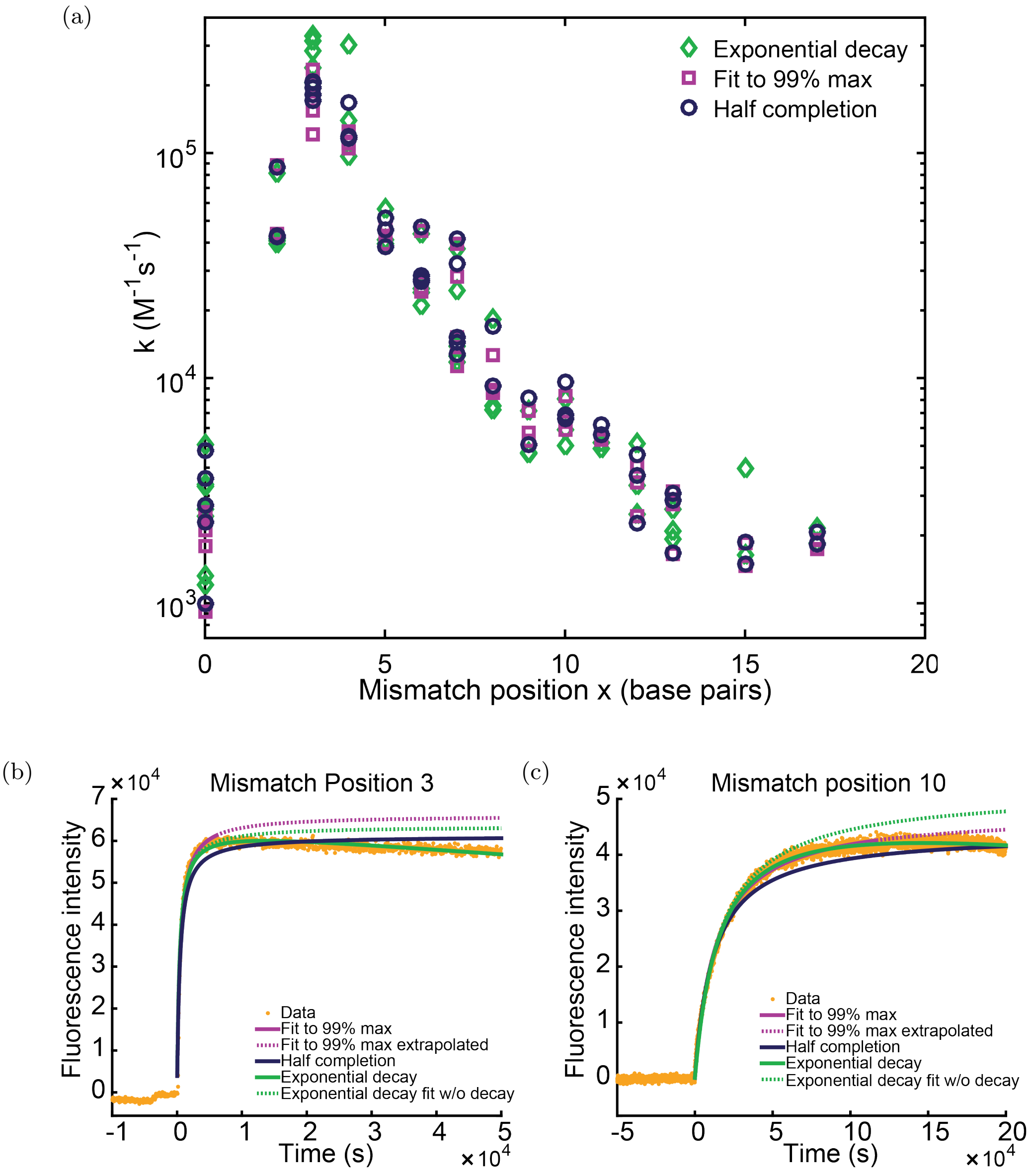

Figure 14: Comparison of fitting methods to determine second-order rate constant k. 14a) k determined from the same individual experiments in the same data sets by three different methods: second-order kinetics fit with exponential decay envelope (to allow for bleaching of the reporter dye) (green diamond), second-order kinetics fit for all points up to the first point exceeding 99% the maximum fluorescence (purple squares) and k derived from time to reach half the maximum recorded fluorescence value (blue circles). Position 0 is the case with no mismatch elimination. All fitting methods give the same non-monotonic relationship between mismatch position and relative reaction rate with maximum at position 3. All fitting methods show a two order of magnitude range of reaction rate constants in agreement with the discussion in the main text. 14b,14c) Comparison of fits to raw data, for example data for mismatch position 3 (14b) and position 10 (14c), using the same three methods. Data points are yellow circles; solid lines show fit results within the range of data used to generate the fits. The purple dotted line shows result of the fit up to 99% peak fluorescence extrapolated to times outside of the fit range. The green dotted line shows a second-order kinetics plot using a and k obtained from the fit using exponential decay envelope (solid green line) for comparison.

Results are shown as purple squares in Supplementary Figure 14a. Example fits over the fitted range are given as solid purple lines in Supplementary Figures 14b and 14c, and dotted purple lines outside of the fitted region. For the data shown in the main text, fit parameters a and k were initiated at $a = f_{\max}$ a.u., $c_0 k = 10^{-3}$ s^{-1} and bounded by $0.9 \times f_{\max} \leq a \leq 1.5 \times f_{\max}$, 0 s^{-1} $\leq c_0 k \leq$1000 s^{-1}.

All three fitting methods produce similar graphs, capturing the non-monotonicity presented in the main text with maximum second-order rate constant for mismatches at position 3, and all methods give an approximate two order of magnitude range of second-order rate constants with changing mismatch position. Discrepancies between methods were similar to discrepancies between separate runs of the same experiment, with typical uncertainties within a factor of 2. We therefore conclude that our conclusions are robust to drift and/or photobleaching during experiments.

3.2 Fitting the kinetics of the two-toehold system

The processing and fitting of the data from the two-toehold system, outlined below, enables us to provide estimates of the rate constants of the individual reactions that give rise to the observed behaviour. These estimates are consistent with our expectations, providing quantitative support for the qualitative interpretation of the experimental behaviour.

Rate equations

The reaction system is modelled by the ODEs:

$$\frac{d[I_5T]}{dt} = k_5[I_5][OT] - k_{-5}[O][I_5T] - k_{53}[I_3][I_5T] + k_{35}[I_5][I_3T] \quad (4)$$

$$\frac{d[I_3T]}{dt} = k_3[I_3][OT] - k_{-3}[O][I_3T] + k_{53}[I_3][I_5T] - k_{35}[I_5][I_3T] \quad (5)$$

We assume no reverse reactions in which output displaces invader from I_5T or I_3T and therefore set $k_{-5} = k_{-3} = 0$. The remaining 4 rate constants k_5, k_3, k_{53}, and k_{35} are parameters to be fit. Time-dependent fluorescence intensities provide measures of $[I_5T](t)$ and $[I_3T](t)$, and the concentrations of other species can be deduced from these and measured initial concentrations $[I_5]_0$, $[I_3]_0$, and $[OT]_0$, as described below.

Deducing concentrations from fluorescence data

Experimental fluorescence traces $f_X(t)$ were obtained for $X = 3, 5$ corresponding to emission wavelengths of Cy3 (I_3 bound to T) and Cy5 (I_5 bound to T), respectively. Simultaneously, fluorescence data for control experiments pre-prepared with saturating levels of T, $f_X^{\text{sat}}(t)$, were obtained. We set $t = 60\,$s at the first data point after mixing (60s is the measured typical mixing time), and define $f_X(0)$ as the last point collected prior to mixing. We define $\bar{f}_X^{\text{sat}}$ as the median raw fluorescence collected for the saturated sample during the experiment.

We normalize and zero our data to obtain an estimate of the fraction of I_X that is bound to T:

$$F_{I_X \text{ bound to} T}(t) = \left(\frac{f_X(t)}{f_X^{\text{sat}}(t)} - \frac{f_X(0)}{f_X^{\text{sat}}(0)} \right) \frac{\bar{f}_X^{\text{sat}}}{\left(\bar{f}_X^{\text{sat}} - f_X(0)\right)} \approx \frac{f_X(t) - f_X(0)}{f_X^{\text{sat}}(t) - f_X(0)}. \quad (6)$$

(This is a good approximation due to the small variation of $f_X^{\text{sat}}(t)$ during the experiment, cf. Supplementary Figures 33-35).

During long fluorometer runs, fluorescence of a species can drift upwards or downwards relative to the control sample used for normalization. The drift is approximately linear on experimental timescales, and small in the experiments reported here (Supplementary Figures

33-35). Nonetheless, the precise values of the populations of invaders bound at long times are important in determining the ratio of fitted parameters k_{35} and k_{53}. A linear fluorescence drift correction is therefore performed. The fluorescence plateau is identified by applying a threshold to the second derivative of normalised fluorescence values smoothed by taking a rolling average of 1000 data points. A median first derivative for all times at which the second derivative is below threshold is used as the gradient m of the drift correction:

$$F^{\text{flattened}}_{I_X \text{ bound to} T}(t) = F_{I_X \text{ bound to} T}(t) - m_X t. \tag{7}$$

There are experimental errors in determining initial concentrations and uncertainties associated with the data processing outlined above, with the result that concentrations deduced from measured initial concentrations and fluorescence data (Supplementary equation 7) do not necessarily satisfy the constraint implicit in our model that 100% of strand T is bound to O at the start of the reaction, and I_3 or I_5 at the end.

We introduce a correction factor c in the determination of concentrations $[I_X T](t)$ such that the experimentally deduced concentrations are approximately consistent with this constraint at large values of t. Specifically, we scale the maximum over the whole experiment of $[I_5 T](t) + [I_3 T](t)$, averaged over a rolling window of 60 data points (∼1 hr), to $[OT]_0$:

$$[OT]_0 = c \times \text{MAX}\left([I_5]_0 \times F^{\text{flattened}}_{I_5 \text{ bound to} T}(t) + [I_3]_0 \times F^{\text{flattened}}_{I_3 \text{ bound to} T}(t)\right) \tag{8}$$

Our final estimates for the concentrations of all species are:

$$\begin{aligned} [I_X T](t) &= c[I_X]_0 \times F^{\text{flattened}}_{I_X \text{ bound to} T}(t) & (9)\\ [I_X](t) &= [I_X]_0 - [I_X T](t) & (10)\\ [OT](t) &= [OT]_0 - [I_5 T](t) - [I_3 T](t) & (11) \end{aligned}$$

Fitting

We solve the ODEs using the MATLAB function `ode15s`. A score is given to a particular set of parameters by calculating a weighted difference between the numerical solution and the normalised data points for $0 < t < t_{\text{stop}}$ where t_{stop} is a user-defined end point.A logarithmic weighting function is used to give greater weight to early time points. The Euclidean lengths of the weighted difference vectors for both data sets (I_5, I_3) are summed to give the score. A prohibitive extra penalty is added if any of the parameters are negative, or if $|\log_{10}(k_{53}/k_{35})| > 3$ i.e. rates cannot be negative and equilibration rates forwards and backwards cannot be more than three orders of magnitude different. The score is minimised using MATLAB function `fminsearch`, using initial values $k_5 = k_3 = k_{53} = k_{35} = 2 \times 10^5$ s^{-1}.

3.3 All fitted curves obtained from single experiments used to produce the experimental points for single-toehold displacement

3.3.1 Position 2

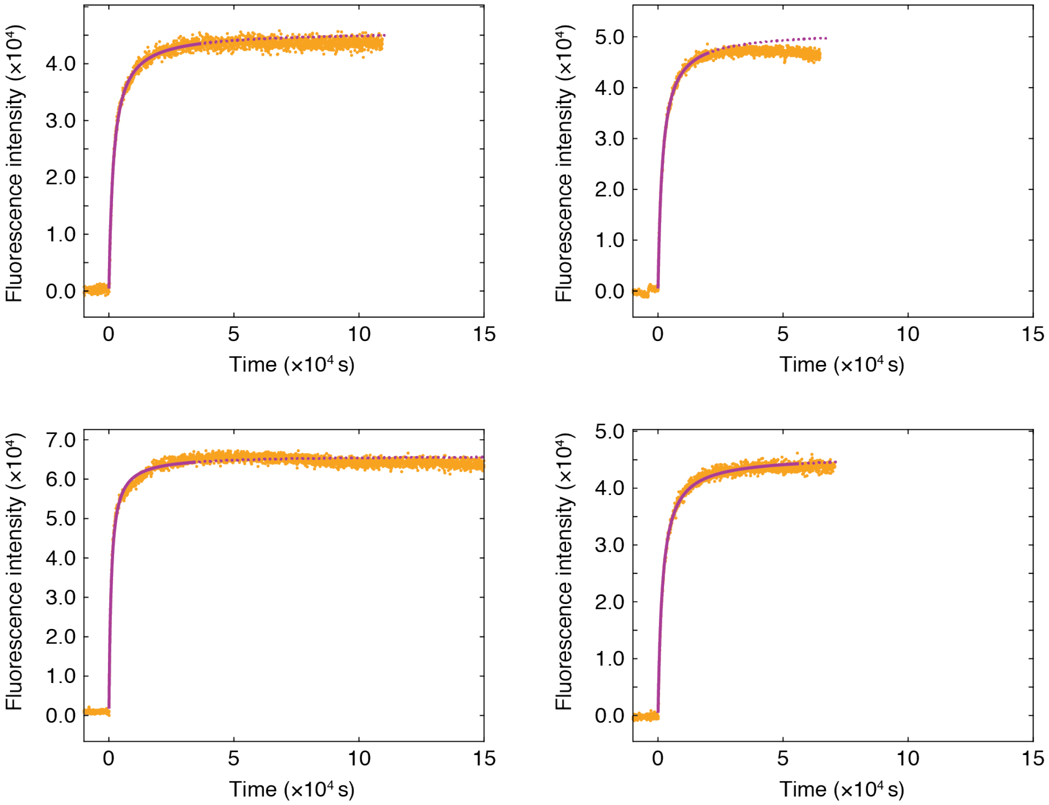

Figure 15: Zeroed fluorescence data for defect position 2 (orange points). Fitted k (corresponding to purple curve) from left to right, top to bottom, in units of $M^{-1}s^{-1}$: 4.30E+04, 4.34E+04, 8.80E+04, 4.38E+04.

3.3.2 Position 3

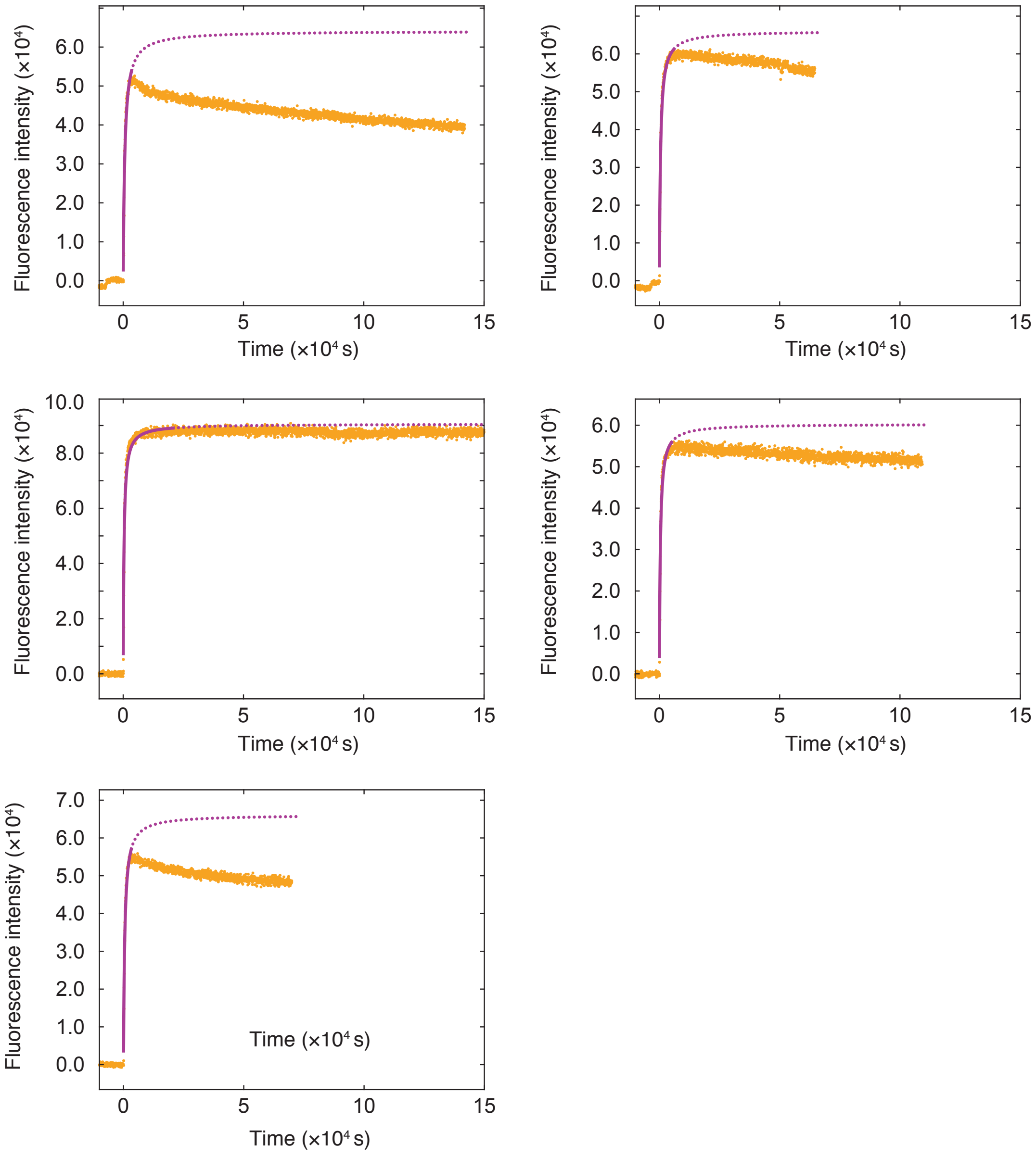

Figure 16: Zeroed fluorescence data for defect position 3 (orange points). Fitted k (corresponding to purple curve) from left to right, top to bottom, in units of $M^{-1}s^{-1}$: 1.21E+05, 1.69E+05, 2.32E+05, 2.00E+05, 1.54E+05.

3.3.3 Position 4

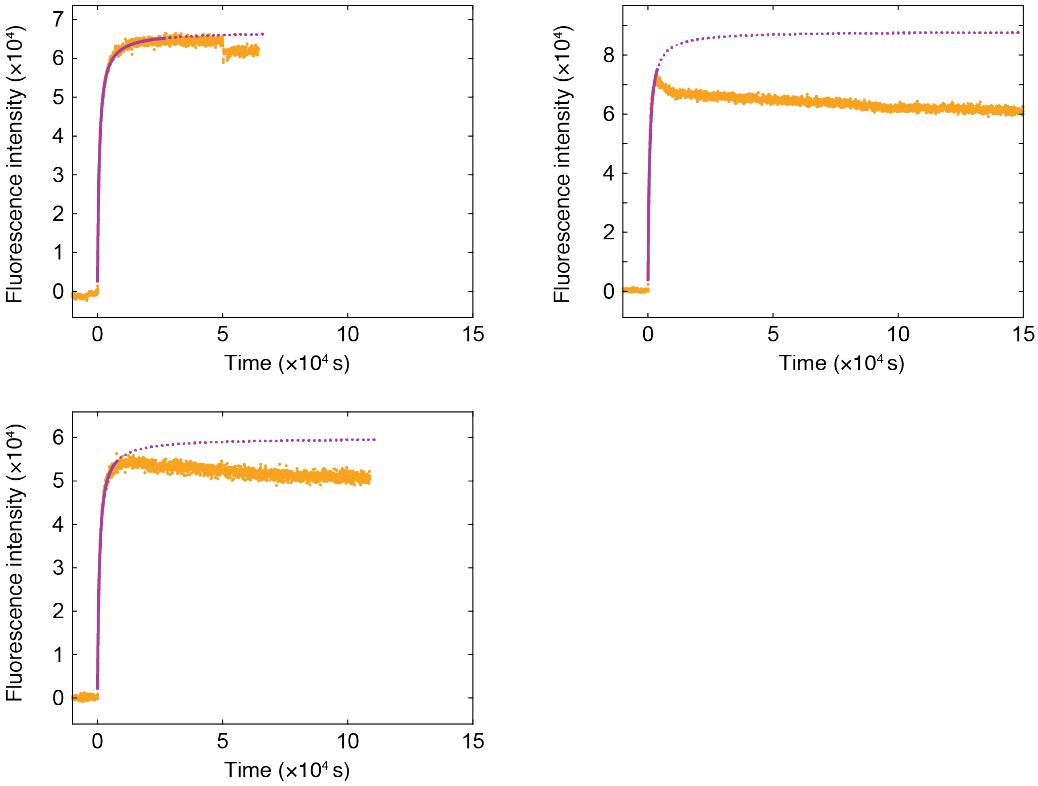

Figure 17: Zeroed fluorescence data for defect position 4 (orange points). Fitted k (corresponding to purple curve) from left to right, top to bottom, in units of $M^{-1}s^{-1}$:1.11E+05, 1.28E+05, 1.05E+05.

3.3.4 Position 5

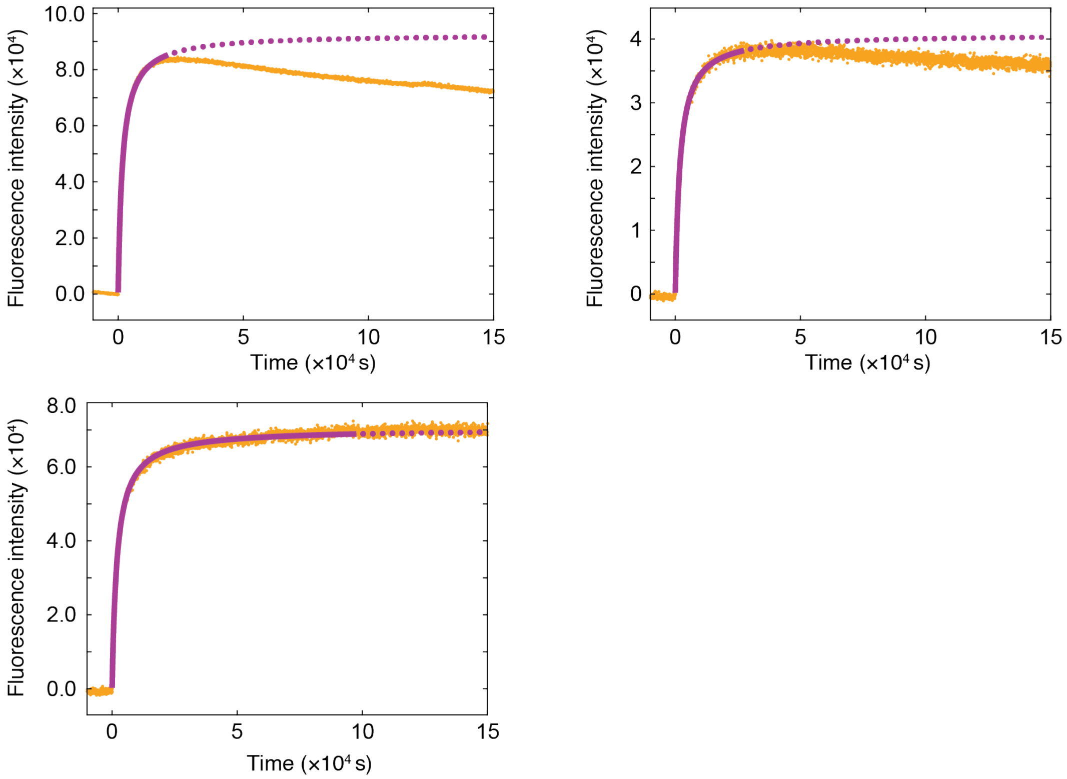

Figure 18: Zeroed fluorescence data for defect position 5 (orange points). Fitted k (corresponding to purple curve) from left to right, top to bottom, in units of $M^{-1}s^{-1}$: 4.59E+04, 4.28E+04, 3.94E+04.

3.3.5 Position 6

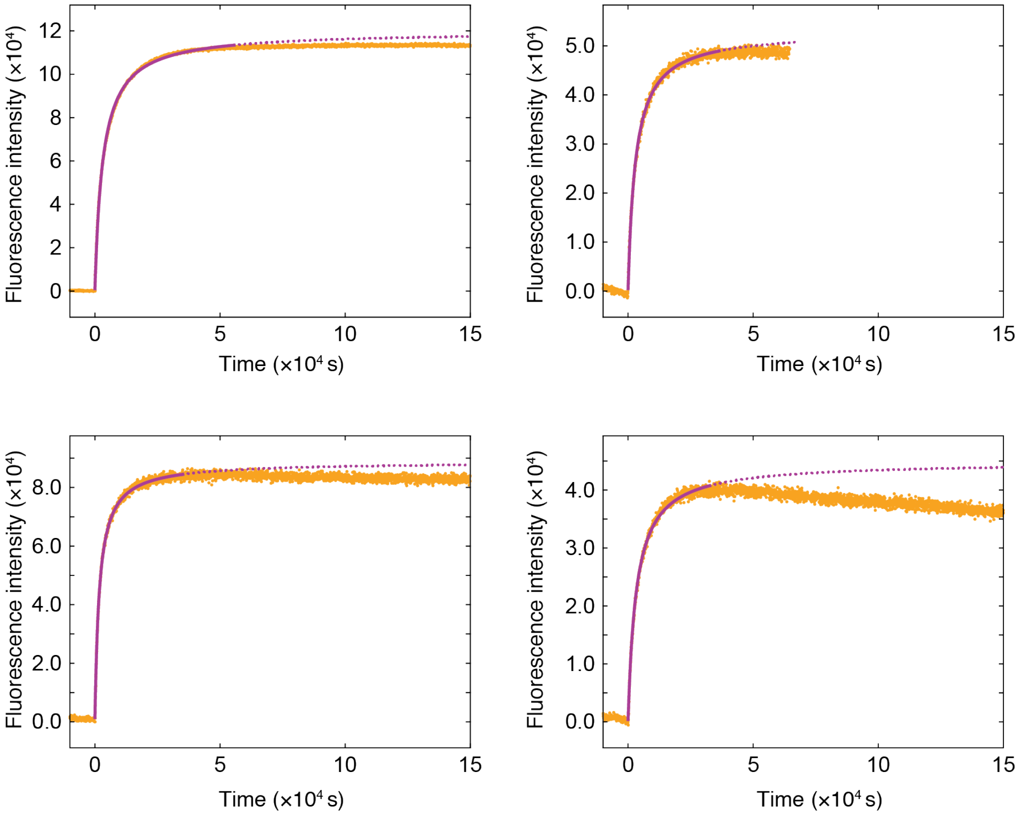

Figure 19: Zeroed fluorescence data for defect position 6 (orange points). Fitted k (corresponding to purple curve) from left to right, top to bottom, in units of $M^{-1}s^{-1}$: 2.52E+04, 2.66E+04, 4.49E+04, 2.42E+04.

3.3.6 Position 7

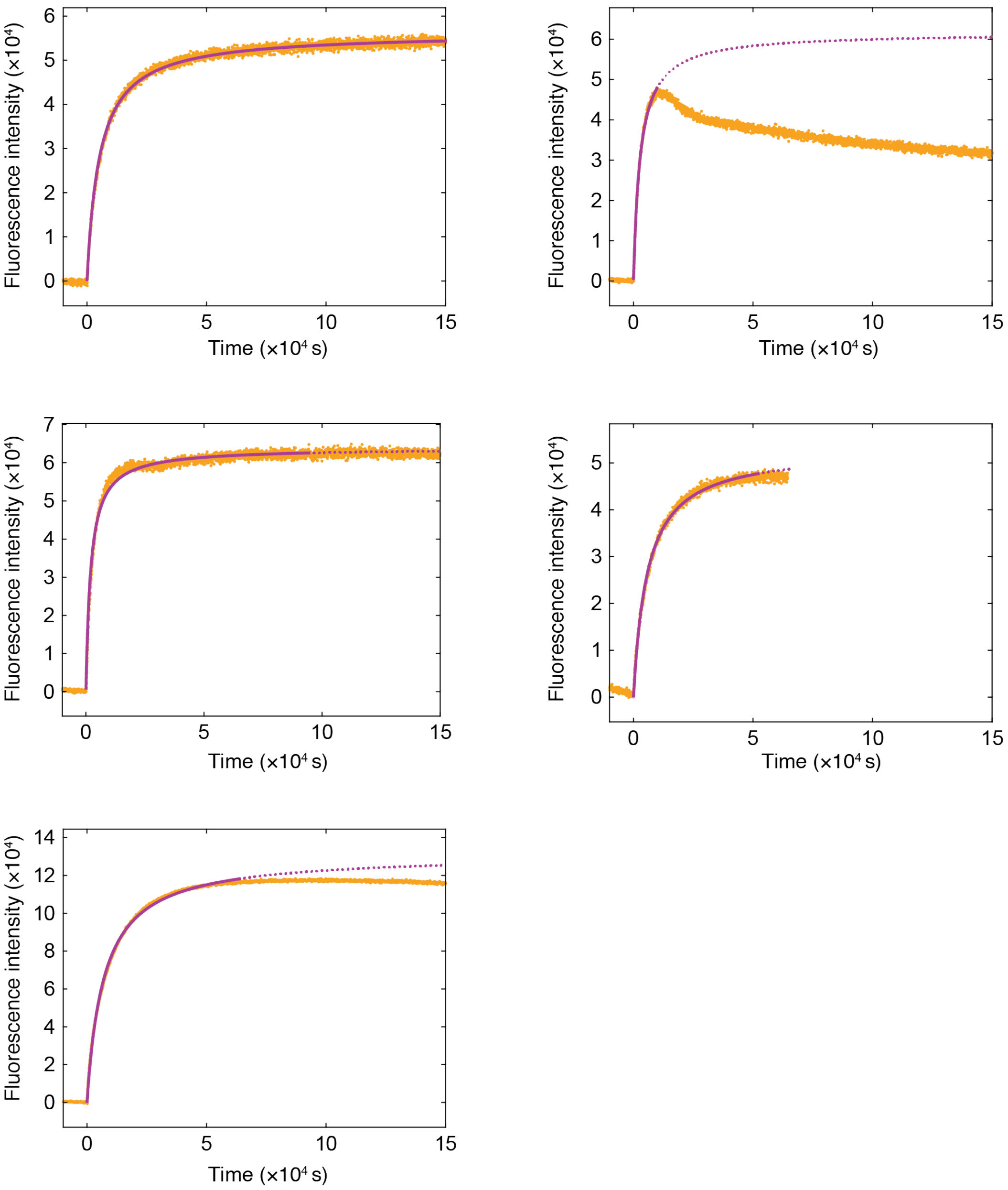

Figure 20: Zeroed fluorescence data for defect position 7 (orange points). Fitted k (corresponding to purple curve) from left to right, top to bottom, in units of $M^{-1}s^{-1}$: 1.52E+04, 2.83E+04, 3.95E+04, 1.38E+04, 1.27E+04.

3.3.7 Position 8

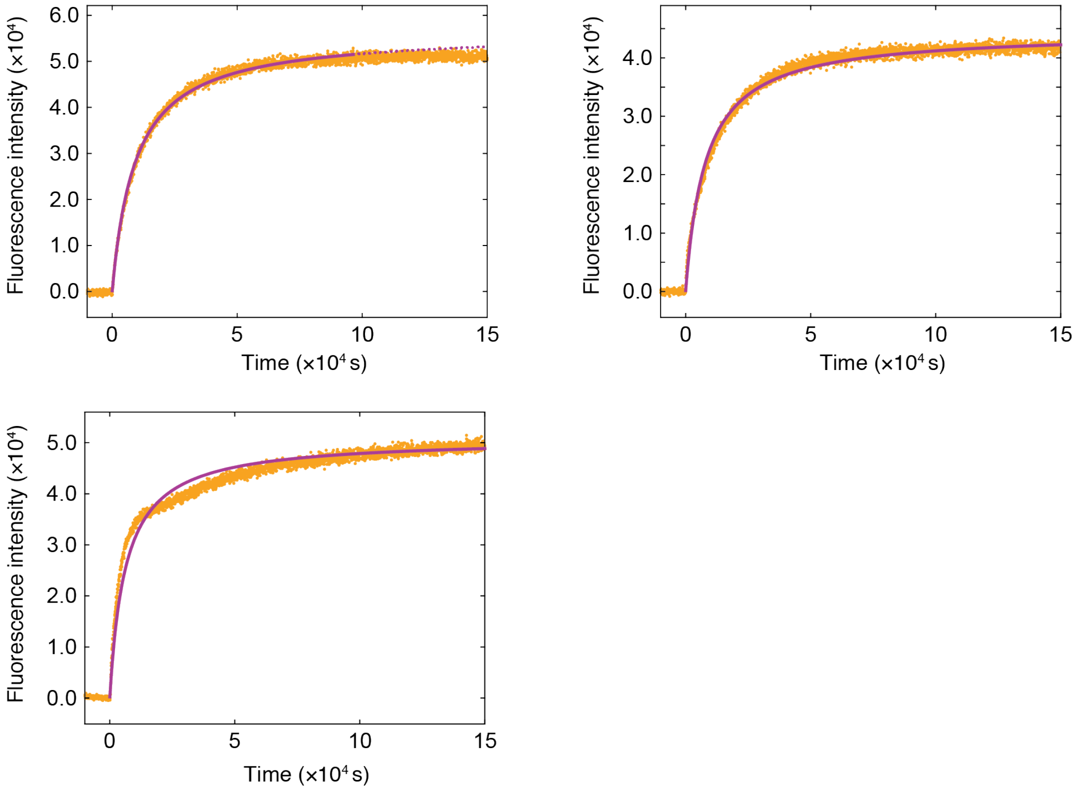

Figure 21: Zeroed fluorescence data for defect position 8 (orange points). Fitted k (corresponding to purple curve) from left to right, top to bottom, in units of $M^{-1}s^{-1}$: 8.58E+03, 9.97E+03, 1.27E+04.

3.3.8 Position 9

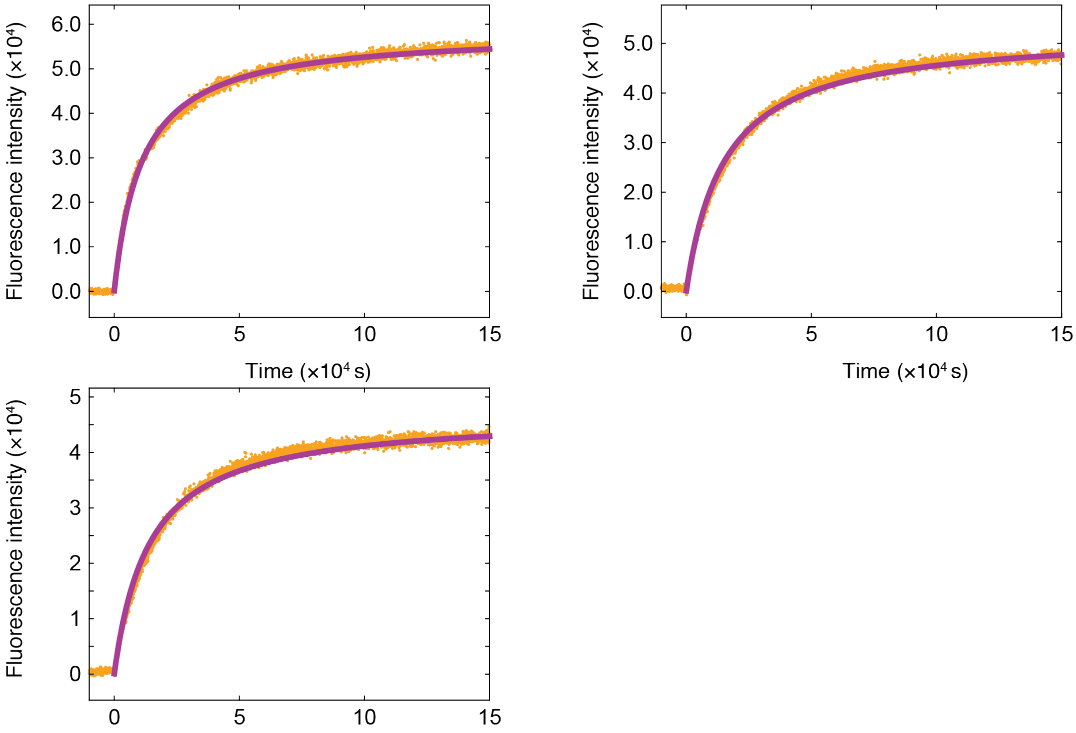

Figure 22: Zeroed fluorescence data for defect position 9 (orange points). Fitted k (corresponding to purple curve) from left to right, top to bottom, in units of $M^{-1}s^{-1}$: 7.19E+03, 5.26E+03, 5.71E+03.

3.3.9 Position 10

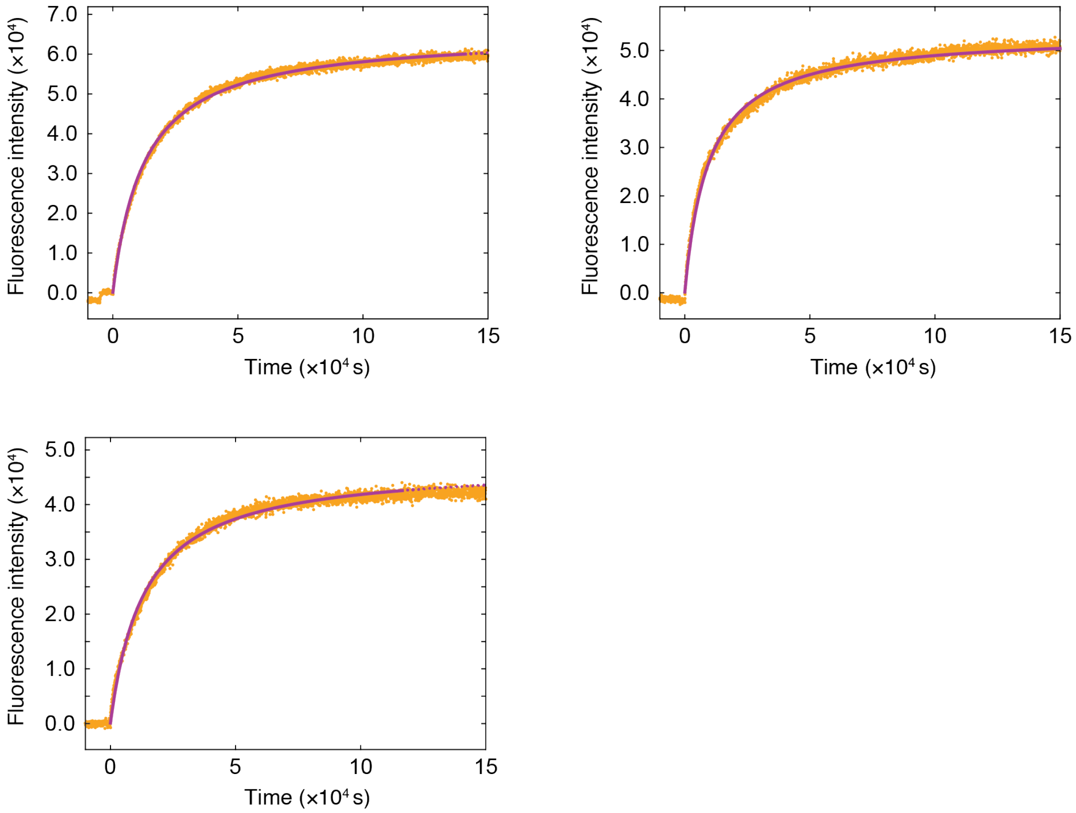

Figure 23: Zeroed fluorescence data for defect position 10 (orange points). Fitted k (corresponding to purple curve) from left to right, top to bottom, in units of $M^{-1}s^{-1}$: 6.38E+03, 8.34E+03, 5.92E+03.

3.3.10 Position 11

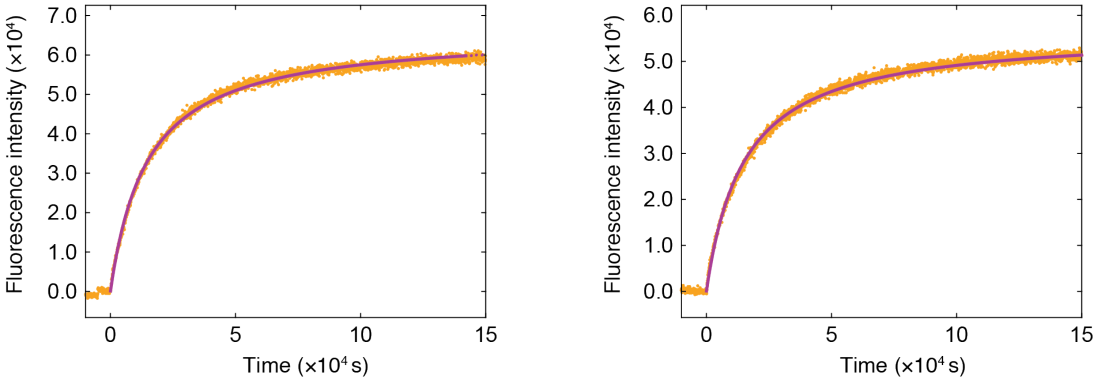

Figure 24: Zeroed fluorescence data for defect position 11 (orange points). Fitted k (corresponding to purple curve) from left to right, top to bottom, in units of $M^{-1}s^{-1}$: 5.55E+03, 5.43E+03.

3.3.11 Position 12

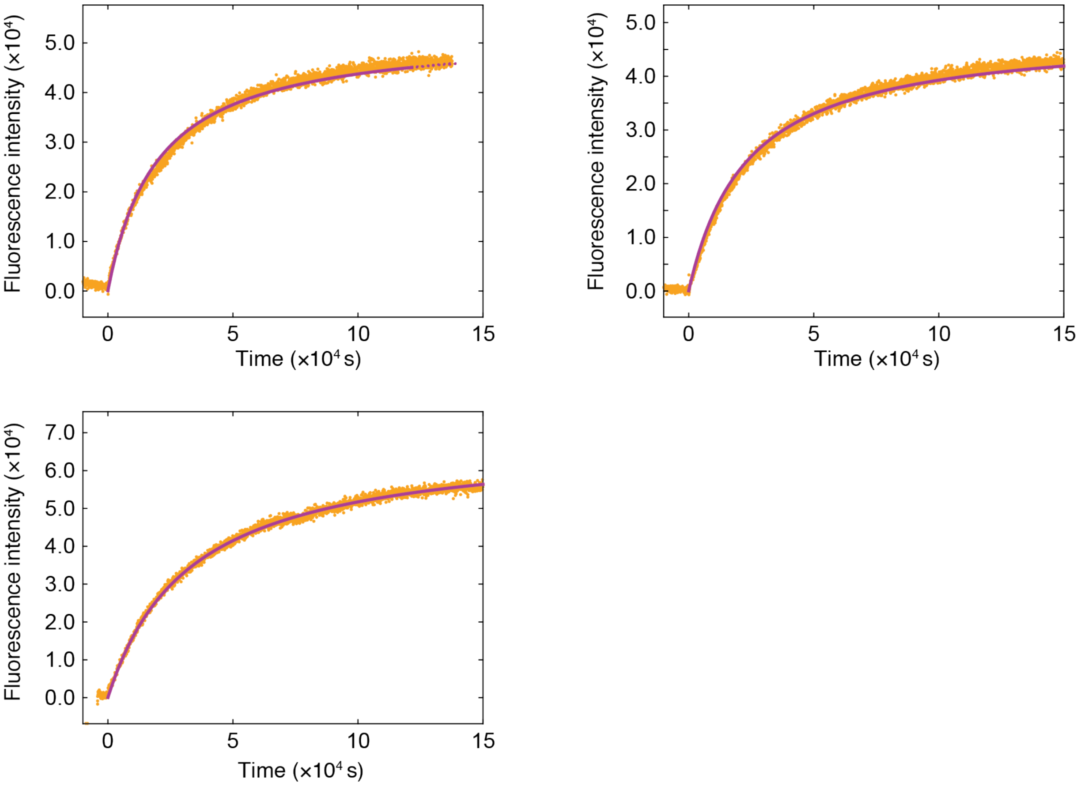

Figure 25: Zeroed fluorescence data for defect position 12 (orange points). Fitted k (corresponding to purple curve) from left to right, top to bottom, in units of $M^{-1}s^{-1}$: 4.05E+03, 3.14E+03, 2.44E+03.

3.3.12 Position 13

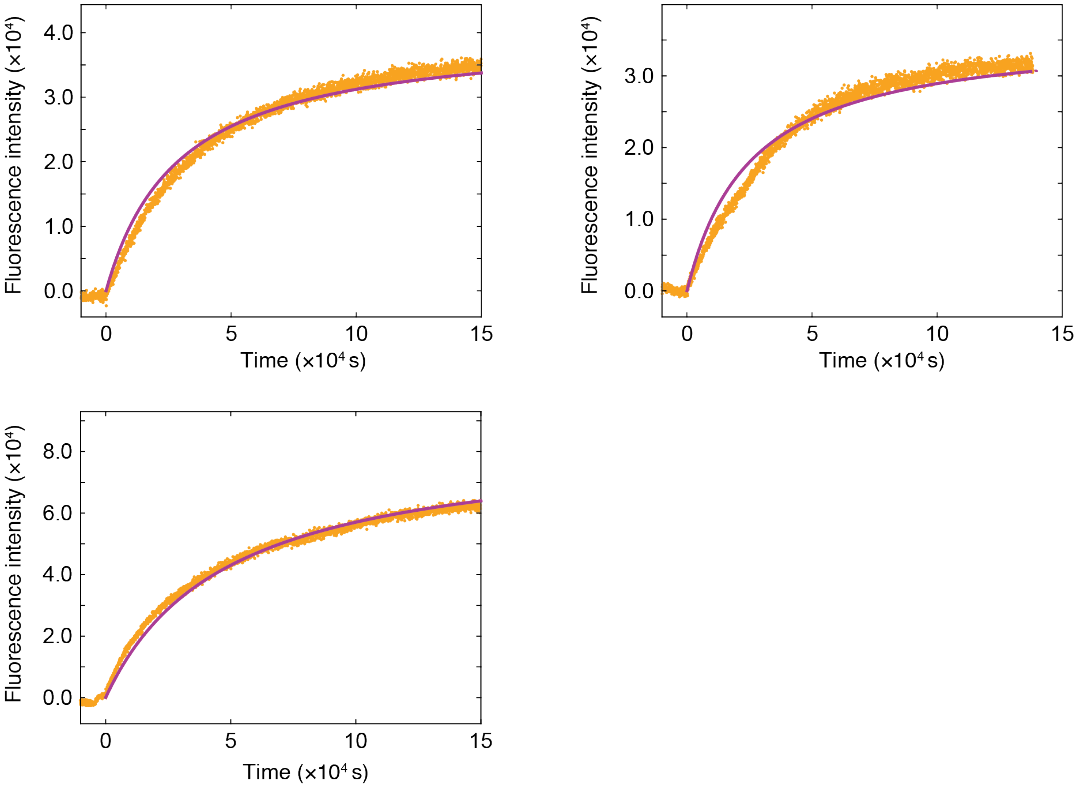

Figure 26: Zeroed fluorescence data for defect position 13 (orange points). Fitted k (corresponding to purple curve) from left to right, top to bottom, in units of $M^{-1}s^{-1}$: 2.76E+03, 3.14E+03, 1.66E+03.

3.3.13 Position 15

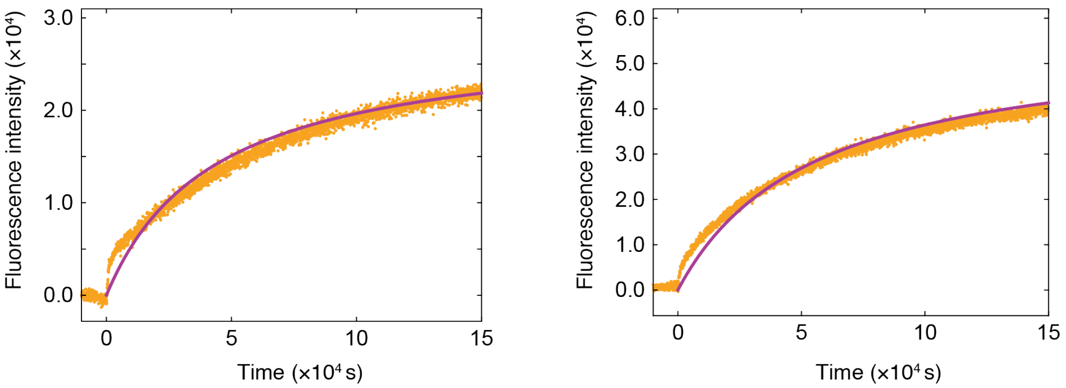

Figure 27: Zeroed fluorescence data for defect position 15 (orange points). Fitted k (corresponding to purple curve) from left to right, top to bottom, in units of $M^{-1}s^{-1}$: 1.81E+03, 1.44E+03.

3.3.14 Position 17

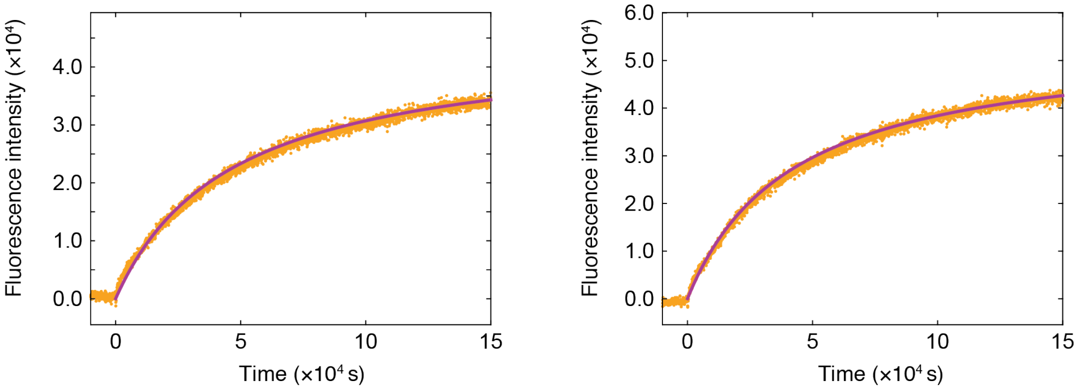

Figure 28: Zeroed fluorescence data for defect position 17 (orange points). Fitted k (corresponding to purple curve) from left to right, top to bottom, in units of $M^{-1}s^{-1}$: 1.73E+03, 1.90E+03.

3.3.15 No Mismatch

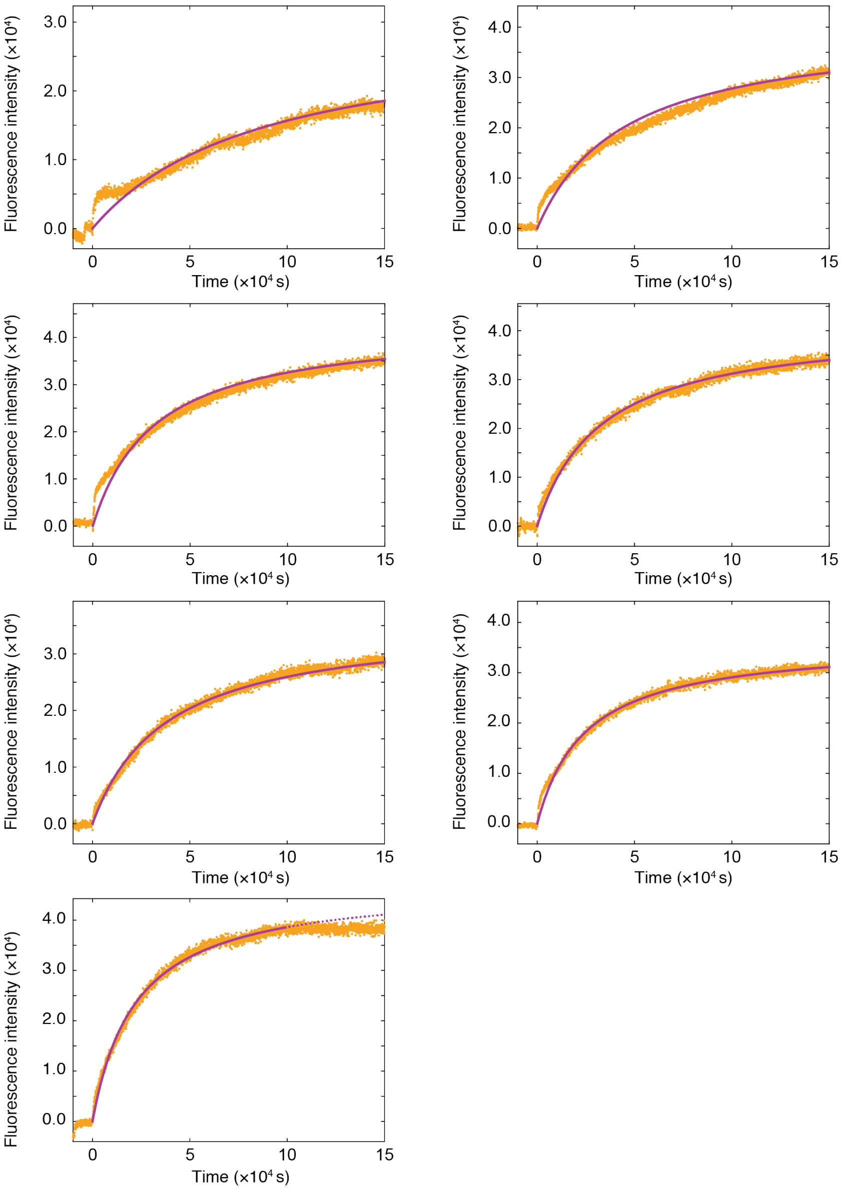

Figure 29: Zeroed fluorescence data for mismatch-free experiemnts (orange points). Fitted k (corresponding to purple curve) from left to right, top to bottom, in units of $M^{-1}s^{-1}$: 1.15E+03, 1.77E+03, 2.52E+03, 2.42E+03, 2.41E+03, 3.29E+03, 4.34E+03, 2.56E+03.

3.4 Unprocessed data for experiments on the minimal catalytic motif

3.4.1 Unprocessed data for experiments monitored with a reporter in real time

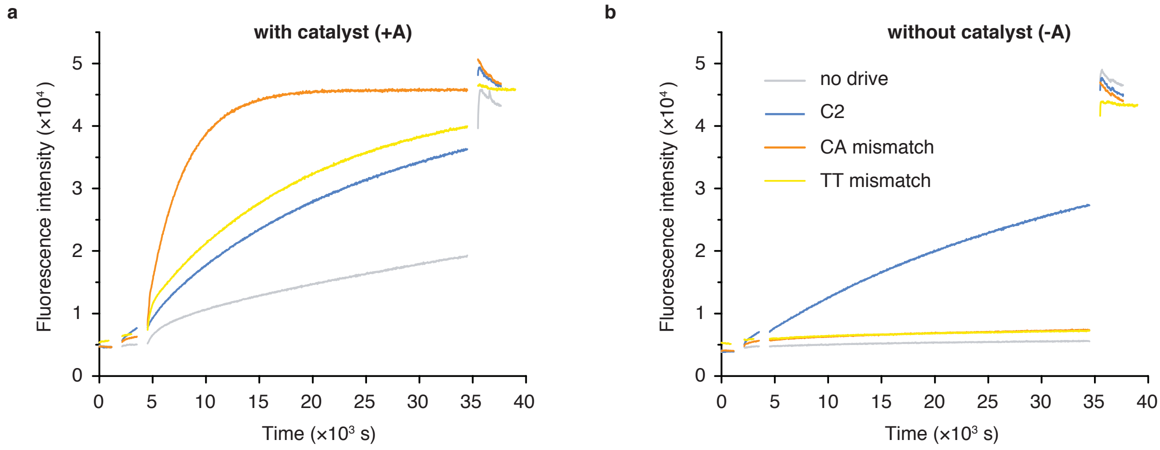

Figure 30: Unprocessed data corresponding to experiments reported in Fig. 5 of the main text. All curves correspond to single experiments. The four separate regions of the plot show fluorescence of the reporter and initial $BD/B_{\rm TT}D/B_{\rm CA}D$ complex in isolation; fluoresnce after the addition of the fuel strand C/C_2, fluorescence after the addition of catalyst $A/A_{\rm TT}/A_{\rm CA}$ (for (a) but not (b)) and fluorescence after the addition of saturating levels of A. An arbitrary time separation of 1000s is used to separate the regions in the plot.

3.4.2 Unprocessed data for experiments involving late addition of the reporter

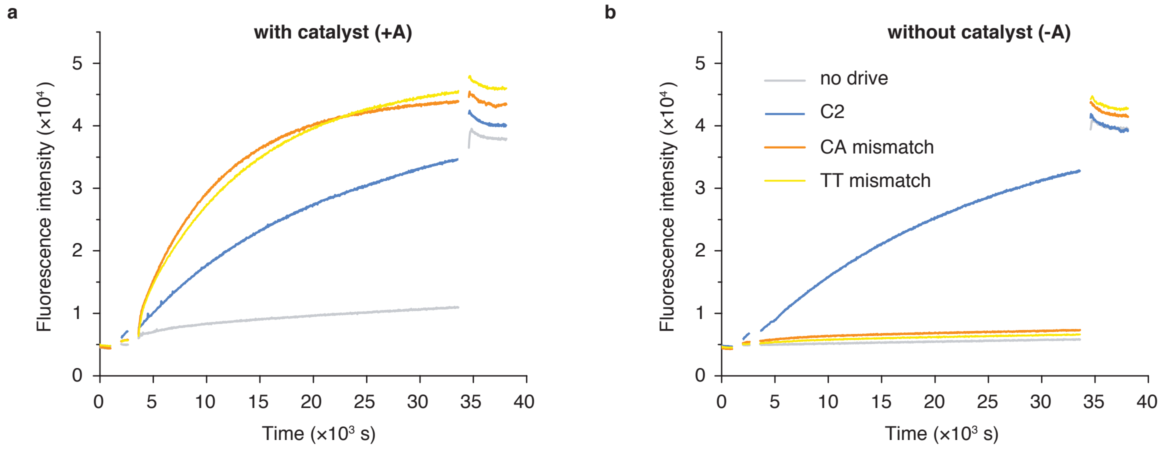

Figure 31: Unprocessed data corresponding to experiments reported in Supplementary Figure 7. All curves correspond to single experiments. The four separate regions of the plot show fluorescence of the reporter and initial $BD/B_{\mathrm{TT}}D/B_{\mathrm{CA}}D$ complex in isolation; fluoresnce after the addition of the fuel strand C/C_2, fluorescence after the addition of catalyst $A/A_{\mathrm{TT}}/A_{\mathrm{CA}}$ (for (a) but not (b)) and fluorescence after the addition of saturating levels of A. An arbitrary time separation of 1000s is used to separate the regions in the plot.

a

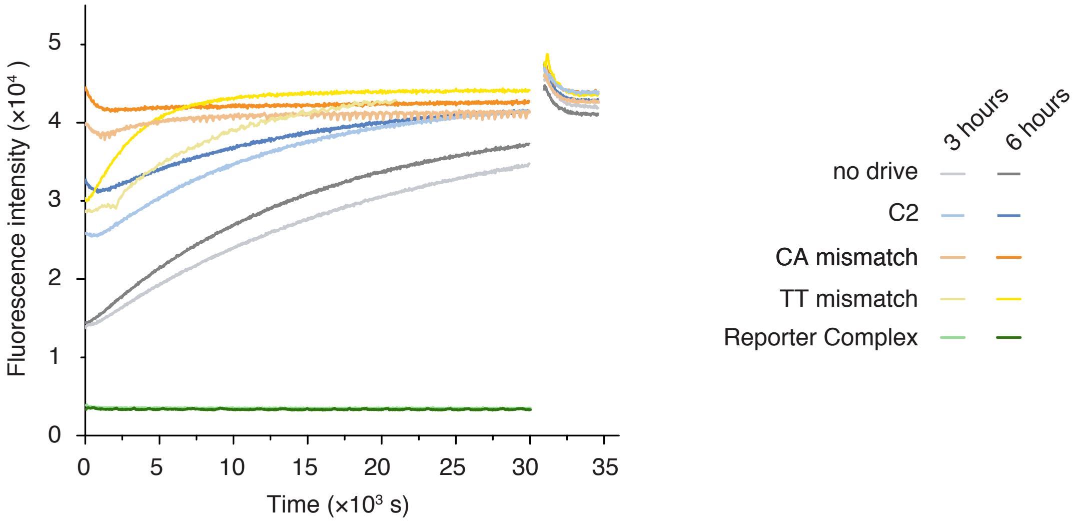

b

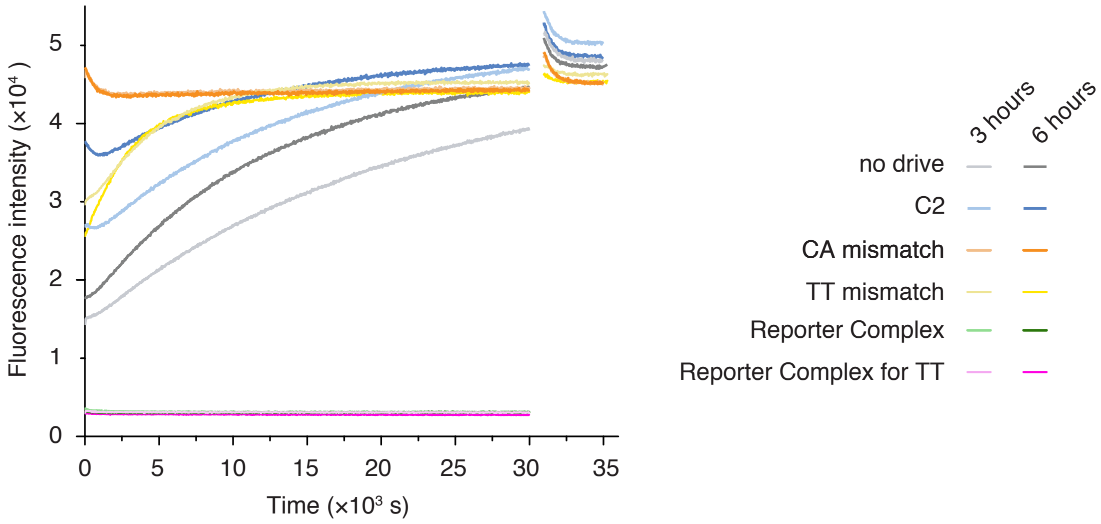

Figure 32: Unprocessed data corresponding to experiments reported in Supplementary Figure 8. All curves correspond to single experiments. Subfigures (a) and (b) correspond to replicas (a) and (b) from Supplementary Figure 8, respectively. The two separate regions in each plot show the data before and after addition of a saturating concentration of strand A. An arbitrary time separation of 1000s is used to separate the regions in the plot.

3.5 Unprocessed data from single experiments for two-toehold displacement

3.5.1 Unprocessed data for two-toehold experiments with mismatches kinetically favouring I_3

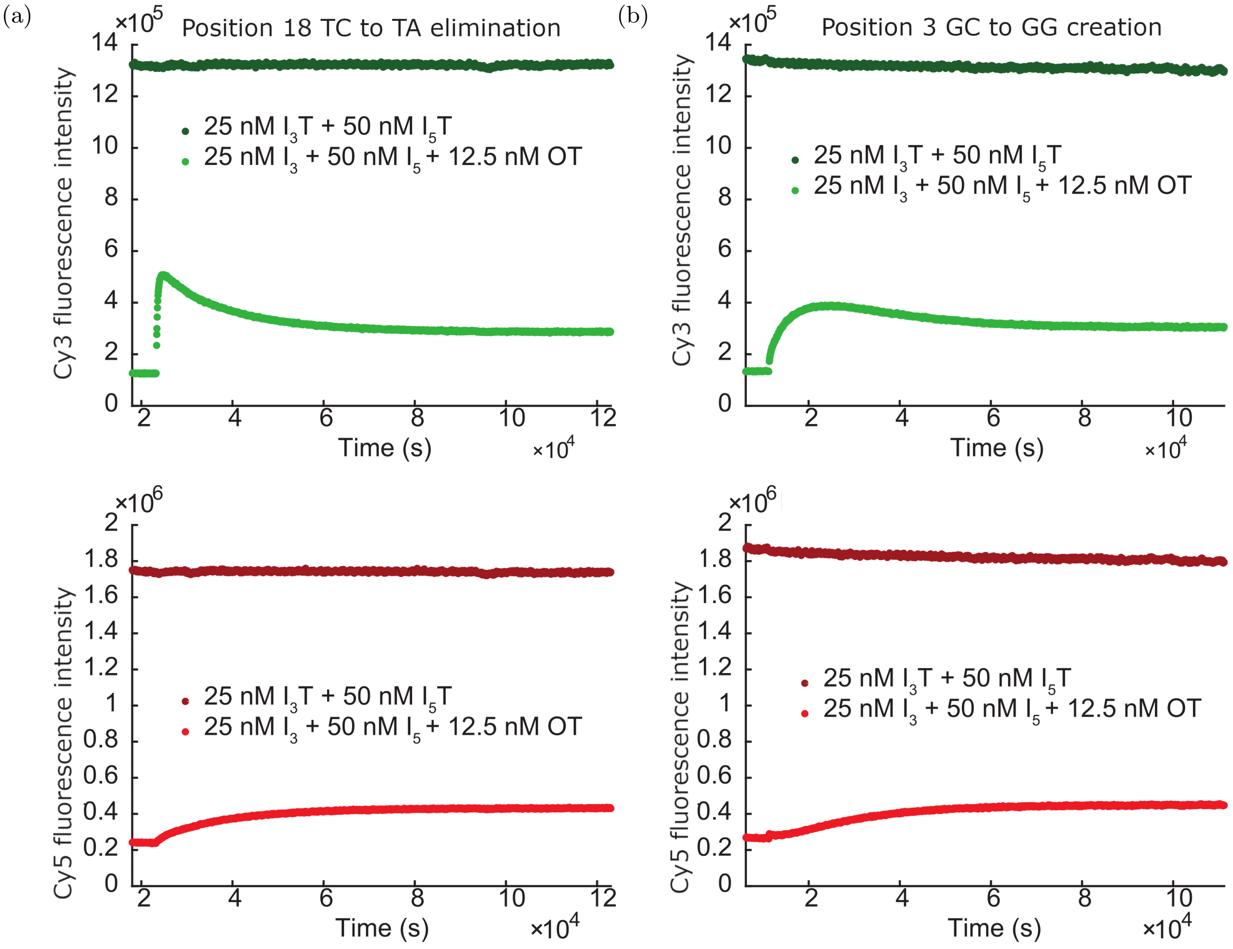

Figure 33: Two-toehold experiments with displacement by I_3 kinetically favoured through mismatch position. Kinetic bias was attempted by T–C →T–A mismatch elimination 18 bp from the 5' toehold (i.e. 3 bp from the 3' toehold) (a) or G–C → G–G mismatch introduction 3 bp from the 5' toehold i.e. 18 bp from the 3' toehold) (b). All reactions have a 2:4:1 ratio of $I_3 : I_5 : OT$ initially present. Data is unprocessed. In each case the lower trace corresponds to addition of OT during observation and the upper trace is a control with the same combination of invaders mixed with excess T.

3.5.2 Unprocessed data for two-toehold experiments with mismatches kinetically favouring I_5

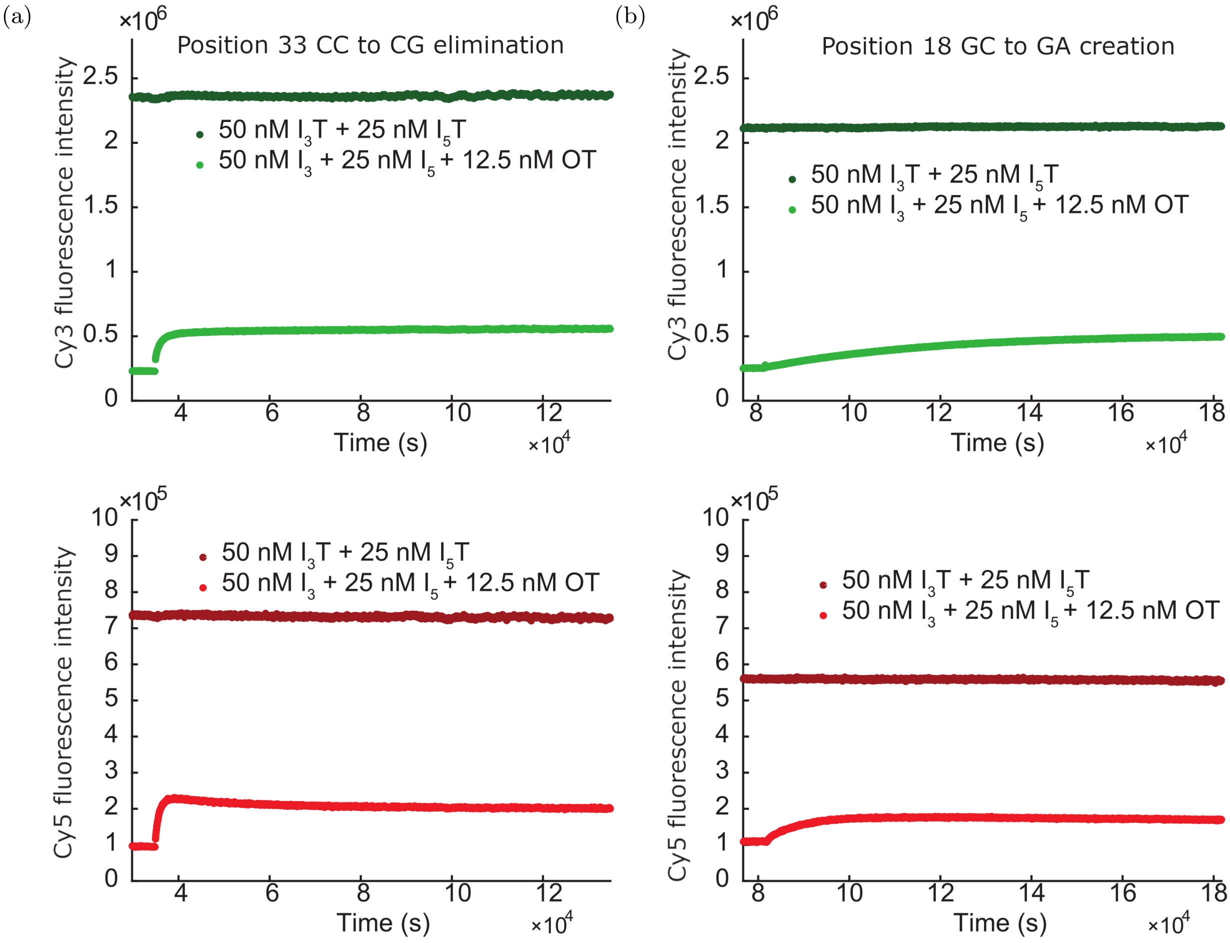

Figure 34: Two-toehold experiments with displacement by I_5 kinetically favoured through mismatch position. Kinetic bias was attempted by C–C →C–G mismatch elimination 3 bp from the 5' toehold (a) or G–C → G–A mismatch introduction 18 bp from the 5' toehold (b). All reactions have a 2:4:1 ratio of $I_5 : I_3 : OT$ initially present. Data is unprocessed. In each case the lower trace corresponds to addition of OT during observation and the upper trace is a control with the same combination of invaders mixed with excess T.

3.5.3 Unprocessed data for mismatch-free invasion in the two-toehold system

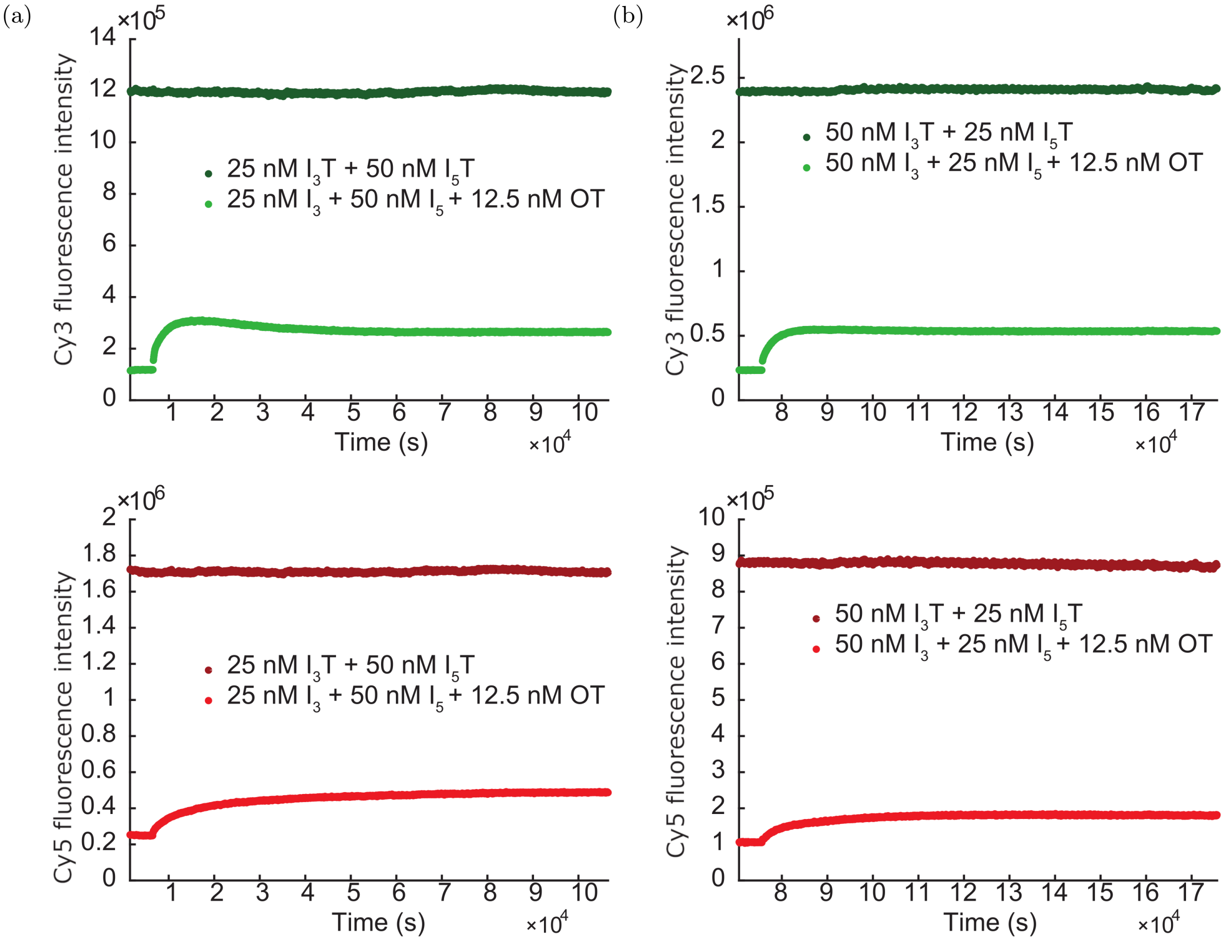

Figure 35: Kinetic data for the two-toehold system in the absence of mismatches. Reactions with a 4:2:1 ratio of $I_5 : I_3 : OT$ (a) or a 2:4:1 ratio of $I_5 : I_3 : OT$ (b). Data is unprocessed. In each case the lower trace corresponds to addition of OT during observation and the upper trace is a control with the same combination of invaders mixed with excess T.

3.5.4 Unprocessed data for the two-toehold system with reversed displacement domains

(a) (b)

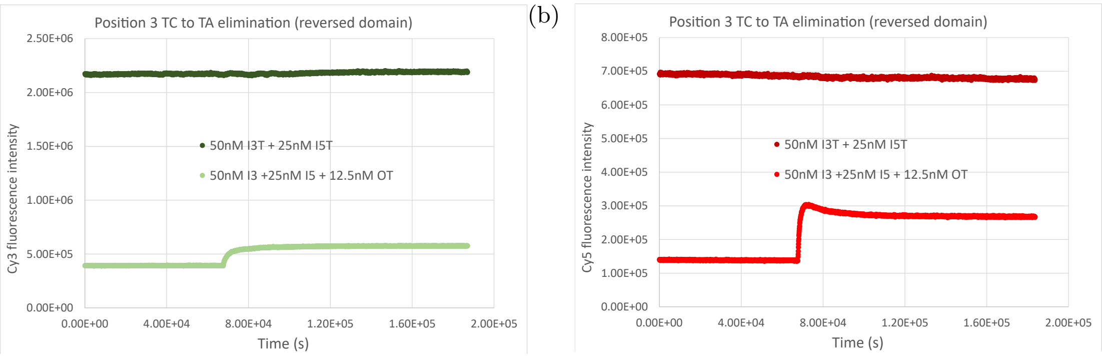

Figure 36: Kinetic data for the two-toehold system with a reversed displacement domain and T–C mismatch elimination at position 3. Reactions initiated with a 2:4:1 ratio of $I_5 : I_3 : OT$; (a) and (b) show Cy3 and Cy5 fluorescence channels, respectively. Data is unprocessed. In each case the lower trace corresponds to addition of OT during observation and the upper trace is a control with the same combination of invaders mixed with excess T.

(a) (b)

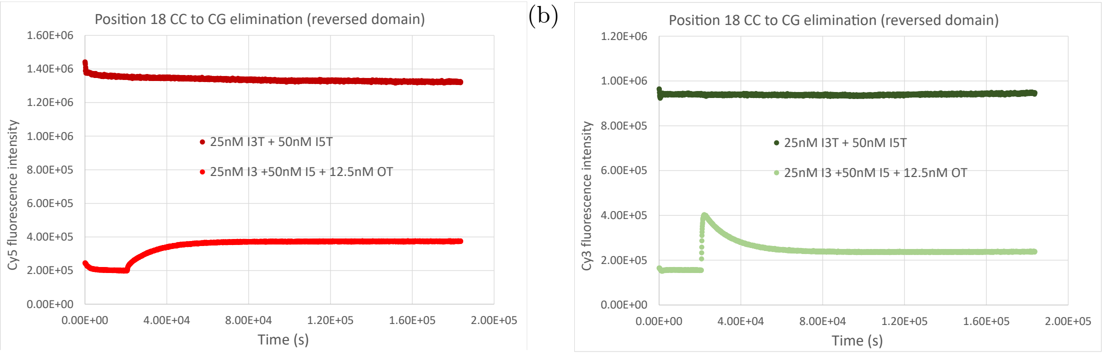

Figure 37: Kinetic data for the two-toehold system with a reversed displacement domain and C–C mismatch elimination at position 18. Reactions initiated with a 4:2:1 ratio of $I_5 : I_3 : OT$; (a) and (b) show Cy3 and Cy5 fluorescence channels, respectively. Data is unprocessed. In each case the lower trace corresponds to addition of OT during observation and the upper trace is a control with the same combination of invaders mixed with excess T.

(a)

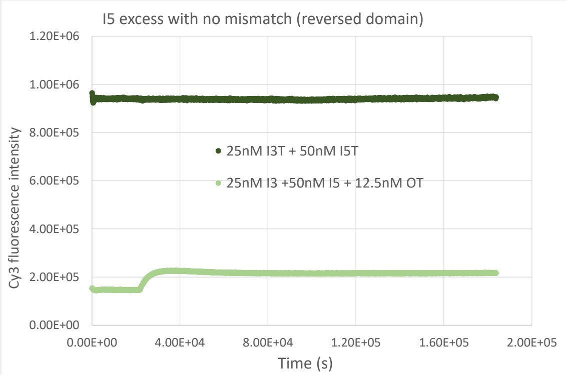

(b)

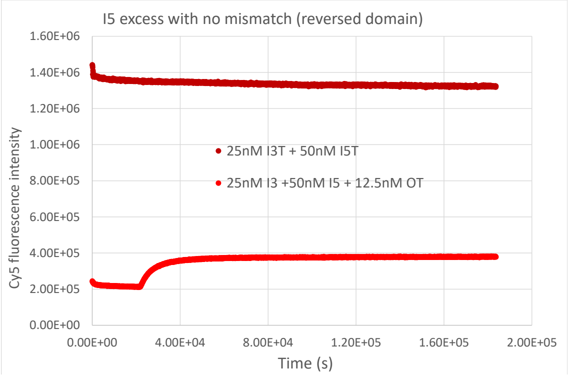

Figure 38: Kinetic data for the two-toehold system with a reversed displacement domain, no mismatch, and an excess of I_5 invaders. Reactions initiated with a 4:2:1 ratio of $I_5 : I_3 : OT$; (a) and (b) show Cy3 and Cy5 fluorescence channels, respectively. Data is unprocessed. In each case the lower trace corresponds to addition of OT during observation and the upper trace is a control with the same combination of invaders mixed with excess T.

(a)

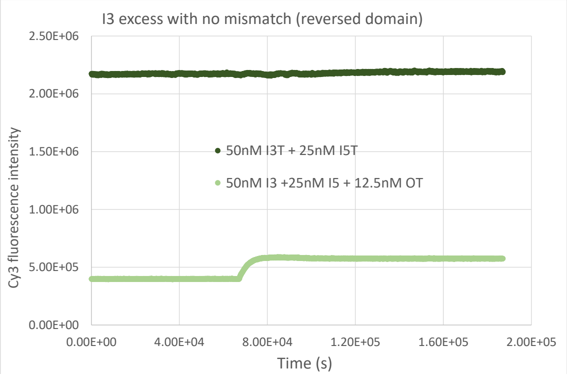

(b)

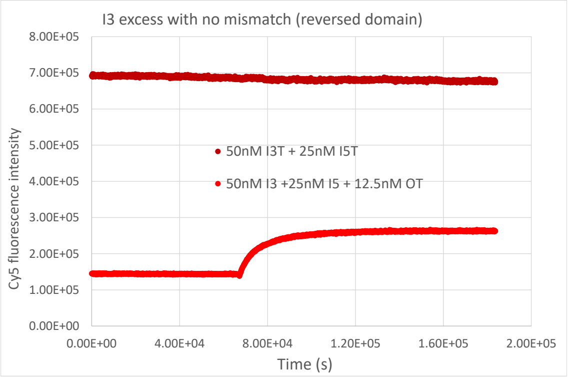

Figure 39: Kinetic data for the two-toehold system with a reversed displacement domain, no mismatch, and an excess of I_3 invaders. Reactions initiated with a 2:4:1 ratio of $I_5 : I_3 : OT$; (a) and (b) show Cy3 and Cy5 fluorescence channels, respectively. Data is unprocessed. In each case the lower trace corresponds to addition of OT during observation and the upper trace is a control with the same combination of invaders mixed with excess T.

4 Supplementary Note 4: Simulation protocols and data

4.1 Forward flux sampling to obtain displacement rates

Forward flux sampling (FFS) allows for the calculation of the flux of trajectories between two local minima in the free-energy landscape, defined by non-overlapping phase space regions A and B. Simultaneously, it provides sample trajectories from which the ensemble of transition pathways can be sampled. In this work, we implement direct FFS.[3,4]

To implement FFS, we define a discrete order parameter Q for the overall displacement transition. The lowest value of Q, $Q = -2$ incorporates the typical states of the system prior to the invader binding to the toehold of the target. The highest value ($Q = Q_{\max} = 9$) incorporates post-displacement states, with the invader bound to the target strand and the output released. The detailed definition of Q is given in Supplementary Table 8.

In an FFS simulation, we first measure the flux of trajectories across the interface λ^0_{-1} between $Q = 0$ and $Q = -1$, $\phi_{\lambda^0_{-1}}$. This flux is defined as the number of times that trajectories cross the interface λ^0_{-1} for the first time since leaving $Q = -2$, divided by the total simulation time, for a long simulation initialised in the $Q = -2$ state.

Having saved the configurations at the interface λ^0_{-1}, we then launch multiple trial trajectories from these points and record the probability of reach the next interface λ^1_0, rather than returning to $Q = -2$. We label this probability $P(\lambda^1_0|\lambda^0_{-1})$, and iterate the procedure for subsequent interfaces, using the successful trajectories at the previous interface as starting points. Overall, the flux from $Q = -2$ to $Q_{\max}$ is obtained as

$$\phi_{Q_{-2}\rightarrow Q_{\max}} = \phi_{\lambda^0_{-1}} \prod_{Q=1}^{Q_{\max}} P(\lambda^Q_{Q-1}|\lambda^{Q-1}_{Q-2}). \tag{12}$$

As previously argued,[1,2] relative fluxes obtained in this way are an accurate estimate of relative second-order rate constants in the oxDNA model, which would be obtained in the low concentration limit, provided any time spent in the intermediate three-stranded complex is not included in the estimate of $\phi_{\lambda^0_{-1}}$. To ensure this correspondence, any rare trajectories that reach $Q = 8$ (the toehold-bound state) during the initial flux simulation are restarted in $Q = -2$.

To obtain relative first order rate constants in the limit of extremely large $[I]$, we simply took the the total number of successful branch migration events initiated from a toehold-only state at λ^8_7, and divide by the total time spent in simulations initiated such states. This is equivalent to repeatedly simulating a OTI complex until an O is displaced, with immediate replacement of I in the toehold-bound state if it detaches from the complex.

4.2 Protocols and detailed results for kinetic measurements

We performed six independent sets of FFS calculations (replicas) for each system. These replicas themselves consisted of multiple parallel simulations in which trial trajectories were launched. Initial flux-calculating simulations were run for an initialisation time to negate the effect of the initial configuration. A summary of the simulations and results at each stage are provided in Supplementary Tables 9 and 10.

To obtain $p(\mathrm{bp}\,x\,\mathrm{reached}|\mathrm{toehold})$ and $p(\mathrm{disp}|\mathrm{bp}\,x\,\mathrm{reached})$ for systems with a mismatch at position x, we simply recorded whether the base pair at position x reached a state with $E < -0.298\,\mathrm{kcal\,mol^{-1}}$ (indicating a well-formed base-pair) during simulations at the final FFS interface. The fraction of simulations in which this was the case directly gives $p(\mathrm{bp}\,x\,\mathrm{reached}|\mathrm{toehold})$, and $p(\mathrm{disp}|\mathrm{bp}\,x\,\mathrm{reached})$ follows from $p(\mathrm{bp}\,x\,\mathrm{reached}|\mathrm{toehold})$ and $p(\mathrm{disp}|\mathrm{toehold})$, the overall success probability at the final FFS interface. These results were obtained in six independent batches of simulations, and standard errors were obtained by comparing these independent estimates.

Q	Distance d/nm	Nearly-formed base pairs n	Base pairs A with $E < E_A$	Base pairs B with $E < E_B$	Correct base pairs B_c with $E < E_B$	Distance d_2/nm
-2	$d > 5.11$	$\sim$	$\sim$	$\sim$	$\sim$	$\sim$
-1	$5.11 > d > 3.41$	$\sim$	$\sim$	$\sim$	$\sim$	$\sim$
0	$3.41 > d > 2.56$	$\sim$	$\sim$	$\sim$	$\sim$	$\sim$
1	$2.56 > d > 1.70$	$\sim$	$\sim$	$\sim$	$\sim$	$\sim$
2	$1.70 > d > 0.852$	$\sim$	$\sim$	$\sim$	$\sim$	$\sim$
3	$0.852 > d$	$n = 0$	$A = 0$	$B = 0$	$\sim$	$\sim$
4	$\sim$	$n > 0$	$A = 0$	$B = 0$	$\sim$	$\sim$
5	$\sim$	$\sim$	$(A > B = 0)$ OR $(A = B = 1)$		$\sim$	$\sim$
6	$\sim$	$\sim$	$\sim$	$1 < B < 4$	$\sim$	$\sim$
7	$\sim$	$\sim$	$\sim$	$B \geq 4$	$B_c < 4$	$\sim$
8	$\sim$	$\sim$	$\sim$	$\sim$	$4 \leq B_c$ & $(B_c < 24$ OR $d_2 < 5.11)$	
9	$\sim$	$\sim$	$\sim$	$\sim$	$B_c = 24$	$d_2 > 5.11$

Table 8: Order parameter definition for FFS simulations of displacement. d is the minimum distance between any base in I and any base in T, measured as the separation of hydrogen-bonding sites. A complementary base pair is defined as nearly formed if the separation of hydrogen bonding sites is ≤ 0.852 nm and at most one of the angular modulating factors that contributes multiplicatively to the hydrogen-bonding energy[5] is zero. A, B and B_c count base pairs between I and T of a sufficient strength; $E_A = -1.43\,\mathrm{kcal\,mol^{-1}}$, and $E_B = -1.79\,\mathrm{kcal\,mol^{-1}}$. Correct base pairs are defined as those intended to form in the finalI/T duplex. d_2 is the minimum distance between any base in O and any base in T. "$\sim$" indicates that no additional restriction is placed on this collective degree of freedom that is not covered by other explicitly stated requirements. Note that this definition of Q assumes that dissociation of the incumbent without displacement is negligible.

To obtain $p(\mathrm{bp}\,x\,\mathrm{reached}|\mathrm{toehold})$ and $p(\mathrm{disp}|\mathrm{bp}\,x\,\mathrm{reached})$ in the mismatch-free case, 1777 new trajectories were launched for the final FFS stage. The invader/substrate base pair that formed (with $E < -0.298$ kcal mol^{-1}) at the greatest distance y from the toehold during simulation was recorded. Any base pair $x \leq y$ (closer to the toehold) was assumed to have been reached in the simulation, allowing a direct calculation of $p(\mathrm{bp}\,x\,\mathrm{reached}|\mathrm{toehold}$. Due to the nature of DNA base-pairing in the oxDNA model, the assumption that all base pairs prior to x will have been formed is sufficiently accurate for our purposes. $p(\mathrm{disp}|\mathrm{bp}\,x\,\mathrm{reached})$ follows from $p(\mathrm{disp}|\mathrm{bp}\,x\,\mathrm{reached})$ and $p(\mathrm{disp}|\mathrm{toehold})$, the overall success probability at the final FFS interface. Random errors were estimated using the standard expression for estimates of binomial probabilities

$$\sigma_p^2 = p(1-p)/N_{\mathrm{sample}}, \tag{13}$$

with N_{sample} the number of trajectories contributing to the estimate of p.

To obtain τ, the average time spent in the three-stranded complex prior to successful displacement, we measured the total simulation time for trajectrories launched from the toehold-bound state at interface λ_7^8. τ is given by dividing this time by the number of successful displacement events. Since trajectories that detach are quickly recorded as failures, and relaunched with a new toehold-bound state, we are effectively exploring the time required for displacement in the first-order limit of a high concentration of invading strands in which the toehold is almost always bound. Data are reported in Supplementary Tables 11 and 12.

Six independent batches of simulations were run. These were collated to obtain τ, and in most cases a standard error could be estimated directly from the spread of the six separate estimates. In the cases with particularly low success rates (mismatch positions 15 and 17, and the mismatch-free system), however, this estimate of the error on τ is problematic. Due to the low absolute number of successful trajectories in these cases, certain batches had much fewer successes than others. This lead to a large apparent variation over batches, which

System	no mismatch	mm2	mm3	mm4
Number of replicas	6	6	6	6
Number of simulations per replica for flux across λ^0_{-1}	10	10	10	10
Initialisation time per simulation $/\mu$s	1.71	1.71	1.71	1.71
Total crossings of λ^0_{-1}	6×10^4	6×10^4	6×10^4	6×10^4
(total time taken$/\mu$s)	585	639	650	640
Average flux across $\lambda^0_{-1}/\mu s^{-1}$	103	93.8	92.3	93.8
Target interface	Total states loaded/successes over all replicas			
λ^1_0	$1.2 \times 10^5/51989$	$1.2 \times 10^5/51813$	$1.2 \times 10^5/51772$	$1.2 \times 10^5/51676$
λ^2_1	$1.2 \times 10^5/61572$	$1.2 \times 10^5/61049$	$1.2 \times 10^5/61380$	$1.2 \times 10^5/60942$
λ^3_2	$1.2 \times 10^5/57738$	$1.2 \times 10^5/57806$	$1.2 \times 10^5/58217$	$1.2 \times 10^5/57342$
λ^4_3	$1.2 \times 10^5/49626$	$1.2 \times 10^5/49103$	$1.2 \times 10^5/48894$	$1.2 \times 10^5/48464$
λ^5_4	$1.8 \times 10^5/18389$	$1.8 \times 10^5/18092$	$1.74 \times 10^5/17058$	$1.8 \times 10^5/17393$
λ^6_5	$9 \times 10^4/29527$	$9 \times 10^4/25931$	$9 \times 10^4/29766$	$9 \times 10^4/28401$
λ^7_6	$5.7 \times 10^4/12963$	$6 \times 10^4/12120$	$6 \times 10^4/14454$	$6 \times 10^4/11187$
λ^8_7	6000/1966	6000/3153	6000/2069	6000/2628
λ^9_8	2409/72	337/134	280/200	388/246
Average flux to displacement$/s^{-1}$	336	5.10×10^3	7.77×10^3	6.31×10^3
Standard error$/s^{-1}$	36.9	488	414	407

Table 9: Results obtained from FFS simulations of displacement for systems with no mismatch, and mismatched up to position 4.

System	mm5	mm7	mm15	mm17
Number of replicas	6	6	6	6
Number of simulations per replica for flux across λ_{-1}^{0}	10	10	10	10
Initialisation time per simulation $/\mu$s	1.71	1.71	1.71	1.71
Total crossings of λ_{-1}^{0} (total time taken/μs)	6×10^4 647	6×10^4 645	6×10^4 634	6×10^4 649
Average flux across $\lambda_{-1}^{0}/\mu\text{s}^{-1}$	92.8	93.0	94.6	92.5
Target interface	Total states loaded/successes over all replicas			
λ_0^1	$1.2 \times 10^5/51837$	$1.2 \times 10^5/52081$	$1.2 \times 10^5/51532$	$1.2 \times 10^5/51791$
λ_1^2	$1.2 \times 10^5/61402$	†	$1.2 \times 10^5/61851$	†
λ_2^3	$1.2 \times 10^5/57432$	$1.2 \times 10^5/29689$	$1.2 \times 10^5/58378$	$1.2 \times 10^5/29519$
λ_3^4	$1.2 \times 10^5/47506$	$1.2 \times 10^5/48564$	$1.2 \times 10^5/47954$	$1.2 \times 10^5/47181$
λ_4^5	$1.8 \times 10^5/16709$	$1.8 \times 10^5/16655$	$1.8 \times 10^5/17435$	$1.8 \times 10^5/16630$
λ_5^6	$9 \times 10^4/28320$	$9 \times 10^4/28510$	$9 \times 10^4/26669$	$9 \times 10^4/27929$
λ_6^7	$5.7 \times 10^4/13134$	$6 \times 10^4/13791$	$6 \times 10^4/10772$	$6 \times 10^4/14361$
λ_7^8	6000/2366	6000/2541	6000/2942	6000/2703
λ_8^9	814/263	1584/170	3121/109	2694/61
Average flux to displacement/s^{-1}	3.17×10^3	1.29×10^3	358	268
Standard error/s^{-1}	178	150	38.4	40.5

Table 10: Results obtained from FFS simulations of displacement for systems with mismatches at position 5 and beyond. † indicates that in these cases, the transition probabilities from interface λ_0^1 to λ_2^3 were calculated directly, rather than via the intermediate interface λ_1^2.

over-represents the true uncertainty by putting too much weight on outlying batches with few successful trajectories when calculating the uncertainty in the mean of the time per success.

In these cases we instead noted that $\tau = \tau' p(\text{disp}|\text{toehold})$, where τ' is the the average length of a trajectory launched from the final interface, whether successful or not. Assuming that relative uncertainty in $p(\text{disp}|\text{toehold})$ dominates the error (due to its low value and the fact that τ' is dominated by the well-sampled time per failed trajectory), we estimated the error $\sigma^2_{p(\text{disp}|\text{toehold})}$ using Supplementary Equation 13. The standard error on τ then follows as $\sigma_\tau = \tau\sigma_{p(\text{disp}|\text{toehold})}/p(\text{disp}|\text{toehold})$. Details of the measurement outcomes are given in Supplementary Tables 11 and 12.

	Attempts/success/total time (μs)			
	no mismatch	2	3	4
Batch 1	327/7/92.9	60/28/144	49/35/168	52/36/111
Batch 2	487/14/137	70/23/129	41/32/127	69/45/155
Batch 3	454/13/137	53/20/101	53/36/154	74/46/175
Batch 4	364/13/116	57/20/103	49/38/165	63/43/151
Batch 5	371/9/121	39/22/105	48/31/125	66/35/140
Batch 6	406/16/129	58/21/117	40/28/137	64/41/140
Aggregate estimate of τ (μs)	10.1	5.22	4.38	3.54
Standard error (μs)	1.2	0.13	0.15	0.13

Table 11: oxDNA estimates of the time per successful displacement reaction in the limit of high invader concentration. Independent batches of simulations are reported for each system, in each case showing the number of trajectories launched from λ_7^8, the number that successfully reached λ_8^9 and the total time.

	Attempts/success/total time (μs)			
	5	7	15	17
Batch 1	150/54/168	331/27/133	543/16/136	471/16/139
Batch 2	122/38/164	309/33/126	480/19/135	434/12/128
Batch 3	133/39/177	229/30/106	563/24/145	471/3/130
Batch 4	127/44/164	305/32/109	545/22/136	528/11/126
Batch 5	181/55/176	219/22/92.2	529/16/135	454/10/111
Batch 6	101/33/146	191/26/82.3	461/12/131	336/9/91.2
Aggregate estimate of τ (μs)	3.77	3.80	7.49	11.9
Standard error (μs)	0.26	0.26	0.71	1.5

Table 12: oxDNA estimates of the time per successful displacement reaction in the limit of high invader concentration. Independent batches of simulations are reported for each system, in each case showing the number of trajectories launched from λ_7^8, the number that successfully reached λ_8^9 and the total time.

4.3 Umbrella sampling to obtain free energies of reaction and free-energy profiles

The rate of convergence of thermodynamic simulations (which seek only to measure relative free energies of macrostates, not dynamical information) can be enhanced by umbrella sampling.[6]

Umbrella involves applying a biasing weight $W(\rho)$ to the sampling procedure to lower free-energy barriers, where ρ is an order parameter for the reaction (which need not be the same order parameter used for FFS). Unbiased averages of any quantity A are then obtained by

$$\langle A \rangle = \frac{\langle A/W(\rho) \rangle_W}{\langle 1/W(\rho) \rangle_W}, \tag{14}$$

where $\langle\rangle_W$ indicates an average over the biased ensemble.

It is often advantageous to split the sampling into several windows focussing on distinct ranges of ρ. Data from overlapping windows can be combined to give the overall free-energy landscape using the Weighted Histogram Analysis Method (WHAM).[7]

4.4 Protocols for thermodynamic sampling

We employed a three-dimensional order parameter $\rho = (\rho_1, \rho_2, \rho_3)$. ρ_1 is the number of correct base pairs between I and T (those that are intended to form in the final state) with energy $E < -0.596$ kcal mol^{-1}. ρ_2 is the number of 'correct' base pairs between O and T (those intended to be present in the initial state) with $E < -0.596$ kcal mol^{-1}. ρ_3 is designed to allow biasing according to the separation of strands, and to encourage branch migration steps by suppressing fraying of strands far from the junction site, relative to fraying at the junction site. Taking d_1^c as the separation of hydrogen-bonding sites of potential correct base pairs between I and T, and d_2^c as the equivalent for O and T, ρ_3 is defined by:

- $\rho_3 = 0$ if $\rho_1 = 0$, $\rho_2 > 0$ and $d_1^c \geq 3.41$ nm.
- $\rho_3 = 1$ if ($\rho_1 = 0$, $\rho_2 > 0$ and $d_1^c < 3.41$ nm) or ($\rho_1 > 0$, $\rho_2 = 0$ and $d_2^c < 3.41$ nm)) or ($\rho_1 > 0$, $\rho_2 > 0$ and the other conditions for $\rho_3 = 2$ are not met).
- $\rho_3 = 2$ if $\rho_1 > 0$, $\rho_2 > 0$ and either (or both) of the base pairs at the toehold end of the invader/substrate duplex and the non-toehold end of the incumbent/substrate duplex have energies $E > -0.596$ kcal mol^{-1} (indicating disruption).
- $\rho_3 = 3$ if $\rho_1 > 0$, $\rho_2 = 0$ and $d_2^c > 3.41$ nm.
- $\rho_3 = 4$ if $\rho_1 = \rho_2 = 0$.

In all simulations, we used a partially-factorised bias $W(\rho) = w(\rho_1, \rho_2)\hat{w}(\rho_3)$.

Simulations were performed in three overlapping windows, with distinct $W(\rho)$:

- Window A: $\rho_1 \leq 4$, $\rho_2 > 0$
- Window B: $\rho_1 > 0$, $\rho_2 > 0$
- Window C: $\rho_1 > 0$, $\rho_2 \leq 3$

No windows included $\rho_1 = \rho_2 = 0$ ($\rho_3 = 4$) states, which are irrelevant to displacement. Simulations were constrained to the windows in question by using $W(\rho) = 0$ for disallowed states. Initially, biasing potentials $W(\rho)$ were based on prior experience and then optimized by hand following exploratory simulations. The final forms used to obtain the data presented here are given in Supplementary Tables 13-22.

For each system, six independent data production simulations were performed for each window. Individual simulations were run for 4×10^{10} VMMC steps, following a burn-in period of 4×10^8 VMMC steps in which no data were recorded. All data were aggregated and analysed using WHAM to produce Figure 4 in the main text. Note that for clarity this figure presents a profile restricted to the states in which both invader and incumbent have base pairs with the substrate. To estimate statistical error, the six independent simulations at each window

ρ_1 \ ρ_2	0	1	2	3	4	5	6	7	8	9	10	11	12	13	14	15	16	17	18	19	20
0	0											0									
1	0											7000									
2	0											500									
3	0											40									
4	0											3									
5	0											0									
6	0											0									
7	0											0									
8	0											0									
9	0											0									
10	0											0									
11	0											0									
12	0											0									
13	0											0									
14	0											0									
15	0											0									
16	0											0									
17	0											0									
18	0											0									
19	0											0									
20	0											0									
21	0											0									
22	0											0									
23	0											0									
24	0											0									

Table 13: Umbrella biasing weight $W(\rho) = w(\rho_1, \rho_2)\hat{w}(\rho_3)$ used for simulating all systems within window A. The table shows $w(\rho_1, \rho_2)$; in addition, $w(\hat{\rho_3}) = \{0.025, 1, 1, 0, 0\}$ for $\rho_3 = \{0, 1, 2, 3, 4\}$. In rows without entries for each column, the central value applies to all $\rho_2 > 0$.

ρ_1 \ ρ_2	0	1	2	3	4	5	6	7	8	9	10	11	12	13	14	15	16	17	18	19	20
0	0										0										
1	0										7000										
2	0										500										
3	0										40										
4	0	30	30	30	30	30	30	30	30	30	30	30	30	30	30	30	30	30	30	30	3
5	0	60	60	60	60	60	60	60	60	60	60	60	60	60	60	60	60	60	60	6	60
6	0	200	200	200	200	200	200	200	200	200	200	200	200	200	200	200	200	200	20	200	200
7	0	200	200	200	200	200	200	200	200	200	200	200	200	200	200	200	200	20	200	200	200
8	0	200	200	200	200	200	200	200	200	200	200	200	200	200	200	200	20	200	200	200	200
9	0	200	200	200	200	200	200	200	200	200	200	200	200	200	200	20	200	200	200	200	200
10	0	200	200	200	200	200	200	200	200	200	200	200	200	200	20	200	200	200	200	200	200
11	0	200	200	200	200	200	200	200	200	200	200	200	200	20	200	200	200	200	200	200	200
12	0	200	200	200	200	200	200	200	200	200	200	200	20	200	200	200	200	200	200	200	200
13	0	200	200	200	200	200	200	200	200	200	200	20	200	200	200	200	200	200	200	200	200
14	0	200	200	200	200	200	200	200	200	200	20	200	200	200	200	200	200	200	200	200	200
15	0	200	200	200	200	200	200	200	200	20	200	200	200	200	200	200	200	200	200	200	200
16	0	200	200	200	200	200	200	200	20	200	200	200	200	200	200	200	200	200	200	200	200
17	0	200	200	200	200	200	200	20	200	200	200	200	200	200	200	200	200	200	200	200	200
18	0	200	200	200	200	200	20	200	200	200	200	200	200	200	200	200	200	200	200	200	200
19	0	200	200	200	200	20	200	200	200	200	200	200	200	200	200	200	200	200	200	200	200
20	0	200	200	200	20	200	200	200	200	200	200	200	200	200	200	200	200	200	200	200	200
21	0	200	200	20	200	200	200	200	200	200	200	200	200	200	200	200	200	200	200	200	200
22	0	200	20	200	200	200	200	200	200	200	200	200	200	200	200	200	200	200	200	200	200
23	0	5	50	50	50	50	50	50	50	50	50	50	50	50	50	50	50	50	50	50	50
24	0										50										

Table 14: Umbrella biasing weight $W(\rho) = w(\rho_1, \rho_2)\hat{w}(\rho_3)$ used for simulating the mismatch-free system within window B. The table shows $w(\rho_1, \rho_2)$; in addition, $w(\hat{\rho_3}) = \{0, 1, 0.05, 0, 0\}$ for $\rho_3 = \{0, 1, 2, 3, 4\}$. In rows without entries for each column, the central value applies to all $\rho_2 > 0$.

ρ_1 \ ρ_2	0	1	2	3	4	5	6	7	8	9	10	11	12	13	14	15	16	17	18	19	20
0	0										0										
1	0										50										
2	0										50										
3	0										20										
4	0	50	50	50	50	50	50	50	50	50	50	50	50	50	50	50	50	6	3	3	3
5	0	100	100	100	100	100	100	100	100	100	100	100	100	100	100	100	10	1	0.3	10	10
6	0	0.2	0.2	0.2	0.2	0.2	0.2	0.2	0.2	0.2	0.2	0.2	0.2	0.2	0.2	0.2	0.2	0.2	0.05	0.2	0.2
7	0	0.2	0.2	0.2	0.2	0.2	0.2	0.2	0.2	0.2	0.2	0.2	0.2	0.2	0.2	0.2	0.2	0.05	0.2	0.2	0.2
8	0	0.2	0.2	0.2	0.2	0.2	0.2	0.2	0.2	0.2	0.2	0.2	0.2	0.2	0.2	0.2	0.05	0.2	0.2	0.2	0.2
9	0	0.2	0.2	0.2	0.2	0.2	0.2	0.2	0.2	0.2	0.2	0.2	0.2	0.2	0.2	0.05	0.2	0.2	0.2	0.2	0.2
10	0	0.2	0.2	0.2	0.2	0.2	0.2	0.2	0.2	0.2	0.2	0.2	0.2	0.2	0.05	0.2	0.2	0.2	0.2	0.2	0.2
11	0	0.2	0.2	0.2	0.2	0.2	0.2	0.2	0.2	0.2	0.2	0.2	0.2	0.05	0.2	0.2	0.2	0.2	0.2	0.2	0.2
12	0	0.2	0.2	0.2	0.2	0.2	0.2	0.2	0.2	0.2	0.2	0.2	0.05	0.2	0.2	0.2	0.2	0.2	0.2	0.2	0.2
13	0	0.2	0.2	0.2	0.2	0.2	0.2	0.2	0.2	0.2	0.2	0.05	0.2	0.2	0.2	0.2	0.2	0.2	0.2	0.2	0.2
14	0	0.2	0.2	0.2	0.2	0.2	0.2	0.2	0.2	0.2	0.05	0.2	0.2	0.2	0.2	0.2	0.2	0.2	0.2	0.2	0.2
15	0	0.2	0.2	0.2	0.2	0.2	0.2	0.2	0.2	0.05	0.2	0.2	0.2	0.2	0.2	0.2	0.2	0.2	0.2	0.2	0.2
16	0	0.2	0.2	0.2	0.2	0.2	0.2	0.2	0.05	0.2	0.2	0.2	0.2	0.2	0.2	0.2	0.2	0.2	0.2	0.2	0.2
17	0	0.2	0.2	0.2	0.2	0.2	0.2	0.05	0.2	0.2	0.2	0.2	0.2	0.2	0.2	0.2	0.2	0.2	0.2	0.2	0.2
18	0	0.2	0.2	0.2	0.2	0.2	0.05	0.2	0.2	0.2	0.2	0.2	0.2	0.2	0.2	0.2	0.2	0.2	0.2	0.2	0.2
19	0	0.2	0.2	0.2	0.2	0.05	0.2	0.2	0.2	0.2	0.2	0.2	0.2	0.2	0.2	0.2	0.2	0.2	0.2	0.2	0.2
20	0	0.2	0.2	0.2	0.05	0.2	0.2	0.2	0.2	0.2	0.2	0.2	0.2	0.2	0.2	0.2	0.2	0.2	0.2	0.2	0.2
21	0	0.2	0.2	0.05	0.2	0.2	0.2	0.2	0.2	0.2	0.2	0.2	0.2	0.2	0.2	0.2	0.2	0.2	0.2	0.2	0.2
22	0	0.2	0.05	0.2	0.2	0.2	0.2	0.2	0.2	0.2	0.2	0.2	0.2	0.2	0.2	0.2	0.2	0.2	0.2	0.2	0.2
23	0	0.05	0.2	0.2	0.2	0.2	0.2	0.2	0.2	0.2	0.2	0.2	0.2	0.2	0.2	0.2	0.2	0.2	0.2	0.2	0.2
24	0	0.2	0.2	0.2	0.2	0.2	0.2	0.2	0.2	0.2	0.2	0.2	0.2	0.2	0.2	0.2	0.2	0.2	0.2	0.2	0.2

Table 15: Umbrella biasing weight $W(\rho) = w(\rho_1, \rho_2)\hat{w}(\rho_3)$ used for simulating the misimatch position-2 system within window B. The table shows $w(\rho_1, \rho_2)$; in addition, $\widehat{w(\rho_3)} = \{0, 1, 0.05, 0, 0\}$ for $\rho_3 = \{0, 1, 2, 3, 4\}$. In rows without entries for each column, the central value applies to all $\rho_2 > 0$.

ρ_1 \ ρ_2	0	1	2	3	4	5	6	7	8	9	10	11	12	13	14	15	16	17	18	19	20
0	0										0										
1	0										50										
2	0										50										
3	0										20										
4	0	50	50	50	50	50	50	50	50	50	50	50	50	50	50	50	50	50	50	3	3
5	0	100	100	100	100	100	100	100	100	100	100	100	100	100	100	100	10	1	6	10	10
6	0	100	100	100	100	100	100	100	100	100	100	100	100	100	100	10	1	0.3	10	10	10
7	0	0.1	0.1	0.1	0.1	0.1	0.1	0.1	0.1	0.1	0.1	0.1	0.1	0.1	0.1	0.1	0.1	0.03	0.1	0.1	0.1
8	0	0.1	0.1	0.1	0.1	0.1	0.1	0.1	0.1	0.1	0.1	0.1	0.1	0.1	0.1	0.1	0.03	0.1	0.1	0.1	0.1
9	0	0.1	0.1	0.1	0.1	0.1	0.1	0.1	0.1	0.1	0.1	0.1	0.1	0.1	0.1	0.03	0.1	0.1	0.1	0.1	0.1
10	0	0.1	0.1	0.1	0.1	0.1	0.1	0.1	0.1	0.1	0.1	0.1	0.1	0.1	0.03	0.1	0.1	0.1	0.1	0.1	0.1
11	0	0.1	0.1	0.1	0.1	0.1	0.1	0.1	0.1	0.1	0.1	0.1	0.1	0.03	0.1	0.1	0.1	0.1	0.1	0.1	0.1
12	0	0.1	0.1	0.1	0.1	0.1	0.1	0.1	0.1	0.1	0.1	0.1	0.03	0.1	0.1	0.1	0.1	0.1	0.1	0.1	0.1
13	0	0.1	0.1	0.1	0.1	0.1	0.1	0.1	0.1	0.1	0.1	0.03	0.1	0.1	0.1	0.1	0.1	0.1	0.1	0.1	0.1
14	0	0.1	0.1	0.1	0.1	0.1	0.1	0.1	0.1	0.1	0.03	0.1	0.1	0.1	0.1	0.1	0.1	0.1	0.1	0.1	0.1
15	0	0.1	0.1	0.1	0.1	0.1	0.1	0.1	0.1	0.03	0.1	0.1	0.1	0.1	0.1	0.1	0.1	0.1	0.1	0.1	0.1
16	0	0.1	0.1	0.1	0.1	0.1	0.1	0.1	0.03	0.1	0.1	0.1	0.1	0.1	0.1	0.1	0.1	0.1	0.1	0.1	0.1
17	0	0.1	0.1	0.1	0.1	0.1	0.1	0.03	0.1	0.1	0.1	0.1	0.1	0.1	0.1	0.1	0.1	0.1	0.1	0.1	0.1
18	0	0.1	0.1	0.1	0.1	0.1	0.03	0.1	0.1	0.1	0.1	0.1	0.1	0.1	0.1	0.1	0.1	0.1	0.1	0.1	0.1
19	0	0.1	0.1	0.1	0.1	0.03	0.1	0.1	0.1	0.1	0.1	0.1	0.1	0.1	0.1	0.1	0.1	0.1	0.1	0.1	0.1
20	0	0.1	0.1	0.1	0.03	0.1	0.1	0.1	0.1	0.1	0.1	0.1	0.1	0.1	0.1	0.1	0.1	0.1	0.1	0.1	0.1
21	0	0.1	0.1	0.03	0.1	0.1	0.1	0.1	0.1	0.1	0.1	0.1	0.1	0.1	0.1	0.1	0.1	0.1	0.1	0.1	0.1
22	0	0.1	0.03	0.1	0.1	0.1	0.1	0.1	0.1	0.1	0.1	0.1	0.1	0.1	0.1	0.1	0.1	0.1	0.1	0.1	0.1
23	0	0.03	0.1	0.1	0.1	0.1	0.1	0.1	0.1	0.1	0.1	0.1	0.1	0.1	0.1	0.1	0.1	0.1	0.1	0.1	0.1
24	0										0.1										

Table 16: Umbrella biasing weight $W(\rho) = w(\rho_1, \rho_2)\hat{w}(\rho_3)$ used for simulating the misimatch position-3 system within window B. The table shows $w(\rho_1, \rho_2)$; in addition, $\widehat{w(\rho_3)} = \{0, 1, 0.05, 0, 0\}$ for $\rho_3 = \{0, 1, 2, 3, 4\}$. In rows without entries for each column, the central value applies to all $\rho_2 > 0$.

										ρ_2										
ρ_1	0	1	2	3	4	5	6	7	8	9	10	11	12	13	14	15	16	17	18	19	20
0	0										0										
1	0										50										
2	0										50										
3	0										20										
4	0	50	50	50	50	50	50	50	50	50	50	50	50	50	50	50	50	50	50	3	3
5	0	100	100	100	100	100	100	100	100	100	100	100	100	100	100	100	10	1	6	10	10
6	0	100	100	100	100	100	100	100	100	100	100	100	100	100	100	10	1	6	10	10	10
7	0	100	100	100	100	100	100	100	100	100	100	100	100	100	10	1	0.3	10	10	10	10
8	0	0.1	0.1	0.1	0.1	0.1	0.1	0.1	0.1	0.1	0.1	0.1	0.1	0.1	0.1	0.1	0.03	0.1	0.1	0.1	0.1
9	0	0.1	0.1	0.1	0.1	0.1	0.1	0.1	0.1	0.1	0.1	0.1	0.1	0.1	0.1	0.03	0.1	0.1	0.1	0.1	0.1
10	0	0.1	0.1	0.1	0.1	0.1	0.1	0.1	0.1	0.1	0.1	0.1	0.1	0.1	0.03	0.1	0.1	0.1	0.1	0.1	0.1
11	0	0.1	0.1	0.1	0.1	0.1	0.1	0.1	0.1	0.1	0.1	0.1	0.1	0.03	0.1	0.1	0.1	0.1	0.1	0.1	0.1
12	0	0.1	0.1	0.1	0.1	0.1	0.1	0.1	0.1	0.1	0.1	0.1	0.03	0.1	0.1	0.1	0.1	0.1	0.1	0.1	0.1
13	0	0.1	0.1	0.1	0.1	0.1	0.1	0.1	0.1	0.1	0.1	0.03	0.1	0.1	0.1	0.1	0.1	0.1	0.1	0.1	0.1
14	0	0.1	0.1	0.1	0.1	0.1	0.1	0.1	0.1	0.1	0.03	0.1	0.1	0.1	0.1	0.1	0.1	0.1	0.1	0.1	0.1
15	0	0.1	0.1	0.1	0.1	0.1	0.1	0.1	0.1	0.03	0.1	0.1	0.1	0.1	0.1	0.1	0.1	0.1	0.1	0.1	0.1
16	0	0.1	0.1	0.1	0.1	0.1	0.1	0.1	0.03	0.1	0.1	0.1	0.1	0.1	0.1	0.1	0.1	0.1	0.1	0.1	0.1
17	0	0.1	0.1	0.1	0.1	0.1	0.1	0.03	0.1	0.1	0.1	0.1	0.1	0.1	0.1	0.1	0.1	0.1	0.1	0.1	0.1
18	0	0.1	0.1	0.1	0.1	0.1	0.03	0.1	0.1	0.1	0.1	0.1	0.1	0.1	0.1	0.1	0.1	0.1	0.1	0.1	0.1
19	0	0.1	0.1	0.1	0.1	0.03	0.1	0.1	0.1	0.1	0.1	0.1	0.1	0.1	0.1	0.1	0.1	0.1	0.1	0.1	0.1
20	0	0.1	0.1	0.1	0.03	0.1	0.1	0.1	0.1	0.1	0.1	0.1	0.1	0.1	0.1	0.1	0.1	0.1	0.1	0.1	0.1
21	0	0.1	0.1	0.03	0.1	0.1	0.1	0.1	0.1	0.1	0.1	0.1	0.1	0.1	0.1	0.1	0.1	0.1	0.1	0.1	0.1
22	0	0.1	0.03	0.1	0.1	0.1	0.1	0.1	0.1	0.1	0.1	0.1	0.1	0.1	0.1	0.1	0.1	0.1	0.1	0.1	0.1
23	0	0.03	0.1	0.1	0.1	0.1	0.1	0.1	0.1	0.1	0.1	0.1	0.1	0.1	0.1	0.1	0.1	0.1	0.1	0.1	0.1
24	0										0.1										

Table 17: Umbrella biasing weight $W(\rho) = w(\rho_1, \rho_2)\hat{w}(\rho_3)$ used for simulating the misimatch position-4 system within window B. The table shows $w(\rho_1, \rho_2)$; in addition, $\hat{w(\rho_3)} = \{0, 1, 0.05, 0, 0\}$ for $\rho_3 = \{0, 1, 2, 3, 4\}$. In rows without entries for each column, the central value applies to all $\rho_2 > 0$.

										ρ_2										
ρ_1	0	1	2	3	4	5	6	7	8	9	10	11	12	13	14	15	16	17	18	19	20
0	0										0										
1	0										50										
2	0										50										
3	0										20										
4	0	50	50	50	50	50	50	50	50	50	50	50	50	50	50	50	50	50	50	3	3
5	0	100	100	100	100	100	100	100	100	100	100	100	100	100	100	100	10	1	6	10	10
6	0	100	100	100	100	100	100	100	100	100	100	100	100	100	100	10	1	6	10	10	10
7	0	100	100	100	100	100	100	100	100	100	100	100	100	100	10	1	0.3	10	10	10	10
8	0	100	100	100	100	100	100	100	100	100	100	100	100	10	1	0.3	10	10	10	10	10
9	0	0.1	0.1	0.1	0.1	0.1	0.1	0.1	0.1	0.1	0.1	0.1	0.1	0.1	0.1	0.03	0.1	0.1	0.1	0.1	0.1
10	0	0.1	0.1	0.1	0.1	0.1	0.1	0.1	0.1	0.1	0.1	0.1	0.1	0.1	0.03	0.1	0.1	0.1	0.1	0.1	0.1
11	0	0.1	0.1	0.1	0.1	0.1	0.1	0.1	0.1	0.1	0.1	0.1	0.1	0.03	0.1	0.1	0.1	0.1	0.1	0.1	0.1
12	0	0.1	0.1	0.1	0.1	0.1	0.1	0.1	0.1	0.1	0.1	0.1	0.03	0.1	0.1	0.1	0.1	0.1	0.1	0.1	0.1
13	0	0.1	0.1	0.1	0.1	0.1	0.1	0.1	0.1	0.1	0.1	0.03	0.1	0.1	0.1	0.1	0.1	0.1	0.1	0.1	0.1
14	0	0.1	0.1	0.1	0.1	0.1	0.1	0.1	0.1	0.1	0.03	0.1	0.1	0.1	0.1	0.1	0.1	0.1	0.1	0.1	0.1
15	0	0.1	0.1	0.1	0.1	0.1	0.1	0.1	0.1	0.03	0.1	0.1	0.1	0.1	0.1	0.1	0.1	0.1	0.1	0.1	0.1
16	0	0.1	0.1	0.1	0.1	0.1	0.1	0.1	0.03	0.1	0.1	0.1	0.1	0.1	0.1	0.1	0.1	0.1	0.1	0.1	0.1
17	0	0.1	0.1	0.1	0.1	0.1	0.1	0.03	0.1	0.1	0.1	0.1	0.1	0.1	0.1	0.1	0.1	0.1	0.1	0.1	0.1
18	0	0.1	0.1	0.1	0.1	0.1	0.03	0.1	0.1	0.1	0.1	0.1	0.1	0.1	0.1	0.1	0.1	0.1	0.1	0.1	0.1
19	0	0.1	0.1	0.1	0.1	0.03	0.1	0.1	0.1	0.1	0.1	0.1	0.1	0.1	0.1	0.1	0.1	0.1	0.1	0.1	0.1
20	0	0.1	0.1	0.1	0.03	0.1	0.1	0.1	0.1	0.1	0.1	0.1	0.1	0.1	0.1	0.1	0.1	0.1	0.1	0.1	0.1
21	0	0.1	0.1	0.03	0.1	0.1	0.1	0.1	0.1	0.1	0.1	0.1	0.1	0.1	0.1	0.1	0.1	0.1	0.1	0.1	0.1
22	0	0.1	0.03	0.1	0.1	0.1	0.1	0.1	0.1	0.1	0.1	0.1	0.1	0.1	0.1	0.1	0.1	0.1	0.1	0.1	0.1
23	0	0.03	0.1	0.1	0.1	0.1	0.1	0.1	0.1	0.1	0.1	0.1	0.1	0.1	0.1	0.1	0.1	0.1	0.1	0.1	0.1
24	0										0.1										

Table 18: Umbrella biasing weight $W(\rho) = w(\rho_1, \rho_2)\hat{w}(\rho_3)$ used for simulating the misimatch position-5 system within window B. The table shows $w(\rho_1, \rho_2)$; in addition, $\hat{w(\rho_3)} = \{0, 1, 0.05, 0, 0\}$ for $\rho_3 = \{0, 1, 2, 3, 4\}$. In rows without entries for each column, the central value applies to all $\rho_2 > 0$.

										ρ_2										
ρ_1	0	1	2	3	4	5	6	7	8	9	10	11	12	13	14	15	16	17	18	19	20
0	0										0										
1	0										50										
2	0										50										
3	0										20										
4	0	50	50	50	50	50	50	50	50	50	50	50	50	50	50	50	50	50	50	3	3
5	0	100	100	100	100	100	100	100	100	100	100	100	100	100	100	100	100	100	6	10	10
6	0	100	100	100	100	100	100	100	100	100	100	100	100	100	100	100	100	10	10	10	10
7	0	100	100	100	100	100	100	100	100	100	100	100	100	100	100	100	10	10	10	10	10
8	0	100	100	100	100	100	100	100	100	100	100	100	100	100	100	10	10	10	10	10	10
9	0	100	100	100	100	100	100	100	100	100	100	100	100	10	10	10	10	10	10	10	10
10	0	100	100	100	100	100	100	100	100	100	100	10	10	1	10	10	10	10	10	10	10
11	0	0.1	0.1	0.1	0.1	0.1	0.1	0.1	0.1	0.1	0.1	0.1	0.1	0.03	0.1	0.1	0.1	0.1	0.1	0.1	0.1
12	0	0.1	0.1	0.1	0.1	0.1	0.1	0.1	0.1	0.1	0.1	0.1	0.03	0.1	0.1	0.1	0.1	0.1	0.1	0.1	0.1
13	0	0.1	0.1	0.1	0.1	0.1	0.1	0.1	0.1	0.1	0.1	0.03	0.1	0.1	0.1	0.1	0.1	0.1	0.1	0.1	0.1
14	0	0.1	0.1	0.1	0.1	0.1	0.1	0.1	0.1	0.1	0.03	0.1	0.1	0.1	0.1	0.1	0.1	0.1	0.1	0.1	0.1
15	0	0.1	0.1	0.1	0.1	0.1	0.1	0.1	0.1	0.03	0.1	0.1	0.1	0.1	0.1	0.1	0.1	0.1	0.1	0.1	0.1
16	0	0.1	0.1	0.1	0.1	0.1	0.1	0.1	0.03	0.1	0.1	0.1	0.1	0.1	0.1	0.1	0.1	0.1	0.1	0.1	0.1
17	0	0.1	0.1	0.1	0.1	0.1	0.1	0.03	0.1	0.1	0.1	0.1	0.1	0.1	0.1	0.1	0.1	0.1	0.1	0.1	0.1
18	0	0.1	0.1	0.1	0.1	0.1	0.03	0.1	0.1	0.1	0.1	0.1	0.1	0.1	0.1	0.1	0.1	0.1	0.1	0.1	0.1
19	0	0.1	0.1	0.1	0.1	0.03	0.1	0.1	0.1	0.1	0.1	0.1	0.1	0.1	0.1	0.1	0.1	0.1	0.1	0.1	0.1
20	0	0.1	0.1	0.1	0.03	0.1	0.1	0.1	0.1	0.1	0.1	0.1	0.1	0.1	0.1	0.1	0.1	0.1	0.1	0.1	0.1
21	0	0.1	0.1	0.03	0.1	0.1	0.1	0.1	0.1	0.1	0.1	0.1	0.1	0.1	0.1	0.1	0.1	0.1	0.1	0.1	0.1
22	0	0.1	0.03	0.1	0.1	0.1	0.1	0.1	0.1	0.1	0.1	0.1	0.1	0.1	0.1	0.1	0.1	0.1	0.1	0.1	0.1
23	0	0.03	0.1	0.1	0.1	0.1	0.1	0.1	0.1	0.1	0.1	0.1	0.1	0.1	0.1	0.1	0.1	0.1	0.1	0.1	0.1
24	0										0.1										

Table 19: Umbrella biasing weight $W(\rho) = w(\rho_1,\rho_2)\hat{w}(\rho_3)$ used for simulating the misi-match position-7 system within window B. The table shows $w(\rho_1,\rho_2)$; in addition, $w(\hat{\rho_3}) = \{0, 1, 0.05, 0, 0\}$ for $\rho_3 = \{0, 1, 2, 3, 4\}$. In rows without entries for each column, the central value applies to all $\rho_2 > 0$.

										ρ_2										
ρ_1	0	1	2	3	4	5	6	7	8	9	10	11	12	13	14	15	16	17	18	19	20
0	0										0										
1	0										50										
2	0										50										
3	0										20										
4	0	50	50	50	50	50	50	50	50	50	50	50	50	50	50	50	50	50	50	3	3
5	0	100	100	100	100	100	100	100	100	100	100	100	100	100	100	100	100	60	6	10	10
6	0	100	100	100	100	100	100	100	100	100	100	100	100	100	100	100	100	10	10	10	10
7	0	100	100	100	100	100	100	100	100	100	100	100	100	100	100	100	10	10	10	10	10
8	0	100	100	100	100	100	100	100	100	100	100	100	100	100	100	10	10	10	10	10	10
9	0	100	100	100	100	100	100	100	100	100	100	100	100	100	10	10	10	10	10	10	10
10	0	100	100	100	100	100	100	100	100	100	100	100	100	10	10	10	10	10	10	10	10
11	0	100	100	100	100	100	100	100	100	100	100	100	10	10	10	10	10	10	10	10	10
12	0	100	100	100	100	100	100	100	100	100	100	10	10	10	10	10	10	10	10	10	10
13	0	100	100	100	100	100	100	100	100	100	10	10	10	10	10	10	10	10	10	10	10
14	0	100	100	100	100	100	100	100	100	10	10	10	10	10	10	10	10	10	10	10	10
15	0	100	100	100	100	100	100	100	10	10	10	10	10	10	10	10	10	10	10	10	10
16	0	100	100	100	100	20	20	10	10	10	10	10	10	10	10	10	10	10	10	10	10
17	0	100	100	100	10	0.5	10	10	10	10	10	10	10	10	10	10	10	10	10	10	10
18	0	100	100	10	2	0.3	10	10	10	10	10	10	10	10	10	10	10	10	10	10	10
19	0	0.1	0.1	0.1	0.1	0.03	0.1	0.1	0.1	0.1	0.1	0.1	0.1	0.1	0.1	0.1	0.1	0.1	0.1	0.1	0.1
20	0	0.1	0.1	0.1	0.03	0.1	0.1	0.1	0.1	0.1	0.1	0.1	0.1	0.1	0.1	0.1	0.1	0.1	0.1	0.1	0.1
21	0	0.1	0.1	0.03	0.1	0.1	0.1	0.1	0.1	0.1	0.1	0.1	0.1	0.1	0.1	0.1	0.1	0.1	0.1	0.1	0.1
22	0	0.1	0.03	0.1	0.1	0.1	0.1	0.1	0.1	0.1	0.1	0.1	0.1	0.1	0.1	0.1	0.1	0.1	0.1	0.1	0.1
23	0	0.03	0.1	0.1	0.1	0.1	0.1	0.1	0.1	0.1	0.1	0.1	0.1	0.1	0.1	0.1	0.1	0.1	0.1	0.1	0.1
24	0										0.1										

Table 20: Umbrella biasing weight $W(\rho) = w(\rho_1,\rho_2)\hat{w}(\rho_3)$ used for simulating the misi-match position-15 system within window B. The table shows $w(\rho_1,\rho_2)$; in addition, $w(\hat{\rho_3}) = \{0, 1, 0.05, 0, 0\}$ for $\rho_3 = \{0, 1, 2, 3, 4\}$. In rows without entries for each column, the central value applies to all $\rho_2 > 0$.

ρ_1 \ ρ_2	0	1	2	3	4	5	6	7	8	9	10	11	12	13	14	15	16	17	18	19	20
0	0										0										
1	0										50										
2	0										50										
3	0										20										
4	0	50	50	50	50	50	50	50	50	50	50	50	50	50	50	50	50	50	50	3	3
5	0	100	100	100	100	100	100	100	100	100	100	100	100	100	100	100	100	60	6	10	10
6	0	100	100	100	100	100	100	100	100	100	100	100	100	100	100	100	100	10	10	10	10
7	0	100	100	100	100	100	100	100	100	100	100	100	100	100	100	100	10	10	10	10	10
8	0	100	100	100	100	100	100	100	100	100	100	100	100	100	100	10	10	10	10	10	10
9	0	100	100	100	100	100	100	100	100	100	100	100	100	100	10	10	10	10	10	10	10
10	0	100	100	100	100	100	100	100	100	100	100	100	100	10	10	10	10	10	10	10	10
11	0	100	100	100	100	100	100	100	100	100	100	100	10	10	10	10	10	10	10	10	10
12	0	100	100	100	100	100	100	100	100	100	100	10	10	10	10	10	10	10	10	10	10
13	0	100	100	100	100	100	100	100	100	100	10	10	10	10	10	10	10	10	10	10	10
14	0	100	100	100	100	100	100	100	100	10	10	10	10	10	10	10	10	10	10	10	10
15	0	100	100	100	100	100	100	100	10	10	10	10	10	10	10	10	10	10	10	10	10
16	0	100	100	100	100	100	100	10	10	10	10	10	10	10	10	10	10	10	10	10	10
17	0	100	100	100	100	100	10	10	10	10	10	10	10	10	10	10	10	10	10	10	10
18	0	100	100	100	100	10	10	10	10	10	10	10	10	10	10	10	10	10	10	10	10
19	0	100	100	10	10	10	10	10	10	10	10	10	10	10	10	10	10	10	10	10	10
20	0	100	10	1	10	10	10	10	10	10	10	10	10	10	10	10	10	10	10	10	10
21	0	0.05	0.05	0.015	0.05	0.05	0.05	0.05	0.05	0.05	0.05	0.05	0.05	0.05	0.05	0.05	0.05	0.05	0.05	0.05	0.05
22	0	0.05	0.015	0.05	0.05	0.05	0.05	0.05	0.05	0.05	0.05	0.05	0.05	0.05	0.05	0.05	0.05	0.05	0.05	0.05	0.05
23	0	0.015	0.05	0.05	0.05	0.05	0.05	0.05	0.05	0.05	0.05	0.05	0.05	0.05	0.05	0.05	0.05	0.05	0.05	0.05	0.05
24	0										0.05										

Table 21: Umbrella biasing weight $W(\rho) = w(\rho_1, \rho_2)\hat{w}(\rho_3)$ used for simulating the misimatch position-17 system within window B. The table shows $w(\rho_1, \rho_2)$; in addition, $w(\hat{\rho}_3) = \{0, 1, 0.05, 0, 0\}$ for $\rho_3 = \{0, 1, 2, 3, 4\}$. In rows without entries for each column, the central value applies to all $\rho_2 > 0$.

ρ_1 \ ρ_2	0	1	2	3	4	5	6	7	8	9	10	11	12	13	14	15	16	17	18	19	20
0	0	0	0	0	0	0	0	0	0	0	0	0	0	0	0	0	0	0	0	0	0
1	1	30000	30000	30000	0	0	0	0	0	0	0	0	0	0	0	0	0	0	0	0	0
2	1	30000	30000	30000	0	0	0	0	0	0	0	0	0	0	0	0	0	0	0	0	0
3	1	30000	30000	30000	0	0	0	0	0	0	0	0	0	0	0	0	0	0	0	0	0
4	1	30000	30000	30000	0	0	0	0	0	0	0	0	0	0	0	0	0	0	0	0	0
5	1	30000	30000	30000	0	0	0	0	0	0	0	0	0	0	0	0	0	0	0	0	0
6	1	30000	30000	30000	0	0	0	0	0	0	0	0	0	0	0	0	0	0	0	0	0
7	1	30000	30000	30000	0	0	0	0	0	0	0	0	0	0	0	0	0	0	0	0	0
8	1	30000	30000	30000	0	0	0	0	0	0	0	0	0	0	0	0	0	0	0	0	0
9	1	30000	30000	30000	0	0	0	0	0	0	0	0	0	0	0	0	0	0	0	0	0
10	1	30000	30000	30000	0	0	0	0	0	0	0	0	0	0	0	0	0	0	0	0	0
11	1	30000	30000	30000	0	0	0	0	0	0	0	0	0	0	0	0	0	0	0	0	0
12	1	30000	30000	30000	0	0	0	0	0	0	0	0	0	0	0	0	0	0	0	0	0
13	1	30000	30000	30000	0	0	0	0	0	0	0	0	0	0	0	0	0	0	0	0	0
14	1	30000	30000	30000	0	0	0	0	0	0	0	0	0	0	0	0	0	0	0	0	0
15	1	30000	30000	30000	0	0	0	0	0	0	0	0	0	0	0	0	0	0	0	0	0
16	1	30000	30000	30000	0	0	0	0	0	0	0	0	0	0	0	0	0	0	0	0	0
17	1	30000	30000	30000	0	0	0	0	0	0	0	0	0	0	0	0	0	0	0	0	0
18	1	30000	30000	30000	0	0	0	0	0	0	0	0	0	0	0	0	0	0	0	0	0
19	1	30000	30000	30000	0	0	0	0	0	0	0	0	0	0	0	0	0	0	0	0	0
20	1	30000	30000	30000	0	0	0	0	0	0	0	0	0	0	0	0	0	0	0	0	0
21	1	30000	30000	30000	0	0	0	0	0	0	0	0	0	0	0	0	0	0	0	0	0
22	0.1	30000	30000	30000	0	0	0	0	0	0	0	0	0	0	0	0	0	0	0	0	0
23	0.02	30000	30000	30000	0	0	0	0	0	0	0	0	0	0	0	0	0	0	0	0	0
24	0.01	30000	30000	30000	0	0	0	0	0	0	0	0	0	0	0	0	0	0	0	0	0

Table 22: Umbrella biasing weight $W(\rho) = w(\rho_1, \rho_2)\hat{w}(\rho_3)$ used for simulating all systems within window C. The table shows $w(\rho_1, \rho_2)$; in addition, $w(\hat{\rho}_3) = \{0, 1, 1, 0.025, 0\}$ for $\rho_3 = \{0, 1, 2, 3, 4\}$.

were used to construct six independent estimates of the overall free energy change between configurations with 1 and 23 base pairs between I and T, and ≥ 1 base pairs between I and O. These data are reported in Supplementary Table 23, along with data on the overall free energy of displacement (including the initial binding of T and eventual unbinding of O. These final data show that differences in ΔG shown in Fig. 4 of the main text reflect differences in the overall free-energy of displacement.

			$\Delta G/k_\mathrm{B}T$						
	Mismatch position	no mismatch	2	3	4	5	7	15	17
branch migration only	Estimate 1	-4.46	-6.87	-9.27	-9.59	-9.49	-9.09	-10.1	-9.50
	Estimate 2	-3.76	-6.78	-9.22	-8.86	-10.7	-9.26	-9.89	-8.98
	Estimate 3	-4.48	-7.00	-9.23	-9.25	-9.62	-9.19	-9.81	-8.56
	Estimate 4	-4.39	-8.10	-8.57	-8.81	-9.20	-9.87	-9.94	-8.93
	Estimate 5	-5.00	-6.39	-9.73	-9.62	-9.42	-9.74	-9.67	-8.81
	Estimate 6	-4.68	-7.45	-8.55	-8.69	-10.1	-10.0	-10.2	-10.1
	Aggregate estimate	-4.41	-7.13	-9.11	-9.17	-9.74	-9.54	-9.92	-9.07
	Standard error	0.16	0.24	0.19	0.17	0.22	0.16	0.08	0.23
full displacement	Estimate 1	-9.83	-11.6	-14.2	-15.0	-14.9	-13.7	-15.6	-14.5
	Estimate 2	-9.07	-11.6	-13.9	-14.1	-16.3	-14.8	-15.7	-14.7
	Estimate 3	-9.58	-12.4	-13.6	-14.5	-14..6	-14.2	-14.7	-13.4
	Estimate 4	-8.90	-13.1	-13.4	-14.1	-13.9	-15.3	-14.5	-13.3
	Estimate 5	-10.23	-10.9	-15.2	-14.3	-14.9	-14.4	-14.1	-14.2
	Estimate 6	-10.13	-12.8	-13.8	-13.9	-14.7	-15.0	-14.9	-15.4
	Aggregate estimate	-9.61	-12.1	-14.1	-14.4	-14.9	-14.6	-15.0	-14.2
	Standard error	0.22	0.34	0.26	0.16	0.31	0.24	0.25	0.33

Table 23: oxDNA estimates of overall free energies of displacement, showing individual and aggregate estimates, and inferred errors in the aggregate estimate.

References

[1] Srinivas, N., Ouldridge, T. E., Sulc, P., Schaeffer, J. M., Yurke, B., Louis, A. A., Doye, J. P. K., & Winfree, E. "On the biophysics and kinetics of toehold-mediated DNA strand displacement." *Nucleic Acids Research*, **41**:gkt801 (2013). doi:10.1093/nar/gkt801.

[2] Machinek, R. R. F., Ouldridge, T. E., Haley, N. E. C., Bath, J., & Turberfield, A. J. "Programmable energy landscapes for kinetic control of DNA strand displacement." *Nature Communications*, **5**:5324 (2014). doi:10.1038/ncomms6324.

[3] Allen, R. J., Warren, P. B., & ten Wolde, P. R. "Sampling Rare Switching Events in Biochemical Networks." *Phys. Rev. Lett.*, **94**:018104 (2005).

[4] Allen, R. J., Valeriani, C., & ten Wolde, P. R. "Forward flux sampling for rare event simulations." *J. Phys.: Condens. Matter*, **21**:463102 (2009).

[5] Šulc, P., Romano, F., Ouldridge, T. E., Rovigatti, L., Doye, J. P. K., & Louis, A. A. "Sequence-dependent thermodynamics of a coarse-grained DNA model." *The Journal of chemical physics*, **137**:135101 (2012). doi:10.1063/1.4754132.

[6] Torrie, G. M. & Valleau, J. P. "Nonphysical Sampling Distributions in Monte Carlo Free-Energy Estimation: Umbrella Sampling." *J. Comp. Phys.*, **23**:187 (1977).

[7] Kumar, S., Rosenberg, J. M., Bouzida, D., Swendsen, R. H., & Kollman, P. A. "The weighted histogram analysis method for free-energy calculations on biomolecules. I. The method." *J. Comput. Chem.*, **13**:1011 (1992).
